# Study Protocol for Better Evidence for Selecting Transplant Fluids (BEST-Fluids): a pragmatic, registry-based, multi-center, double-blind, randomized controlled trial evaluating the effect of intravenous fluid therapy with Plasma-Lyte 148 versus 0.9% saline on delayed graft function in deceased donor kidney transplantation

**DOI:** 10.1186/s13063-020-04359-2

**Published:** 2020-05-25

**Authors:** Michael G. Collins, Magid A. Fahim, Elaine M. Pascoe, Kathryn B. Dansie, Carmel M. Hawley, Philip A. Clayton, Kirsten Howard, David W. Johnson, Colin J. McArthur, Rachael C. McConnochie, Peter F. Mount, Donna Reidlinger, Laura Robison, Julie Varghese, Liza A. Vergara, Laurence Weinberg, Steven J. Chadban, for the BEST-Fluids Investigators and the Australasian Kidney Trials Network

**Affiliations:** 1 Department of Renal Medicine, Auckland District Health Board, Auckland City Hospital, Auckland, New Zealand; 2 Department of Medicine, Faculty of Medical and Health Sciences, University of Auckland, Auckland, New Zealand; 3 Australasian Kidney Trials Network, The University of Queensland, Brisbane, Australia; 4 Department of Nephrology, Princess Alexandra Hospital, Brisbane, Australia; 5 Australia and New Zealand Dialysis and Transplant (ANZDATA) Registry, South Australian Health and Medical Research Institute (SAHMRI), Adelaide, Australia; 6 Department of Medicine, The University of Adelaide, Adelaide, Australia; 7 Central and Northern Adelaide Renal and Transplantation Service, Royal Adelaide Hospital, Adelaide, Australia; 8 Sydney School of Public Health, Faculty of Medicine and Health, The University of Sydney, Sydney, Australia; 9 Department of Critical Care Medicine, Auckland City Hospital, Auckland, New Zealand; 10 Department of Nephrology, Austin Health, Melbourne, Australia; 11 Department of Medicine (Austin), The University of Melbourne, Parkville, Melbourne, Australia; 12 Department of Anaesthesia, Austin Health, Melbourne, Australia; 13 Department of Surgery, Austin Health, The University of Melbourne, Melbourne, Australia; 14 Department of Renal Medicine, Royal Prince Alfred Hospital, Sydney, Australia; 15 Charles Perkins Centre, The University of Sydney, Sydney, Australia

**Keywords:** Balanced crystalloid, Delayed graft function, End-stage kidney disease, Intravenous fluids, Kidney transplantation, Peri-operative care, Plasma-Lyte 148, Pragmatic trial, Registry trial, Normal saline

## Abstract

**Background:**

Delayed graft function, the requirement for dialysis due to poor kidney function post-transplant, is a frequent complication of deceased donor kidney transplantation and is associated with inferior outcomes and higher costs. Intravenous fluids given during and after transplantation may affect the risk of poor kidney function after transplant. The most commonly used fluid, isotonic sodium chloride (0.9% saline), contains a high chloride concentration, which may be associated with acute kidney injury, and could increase the risk of delayed graft function. Whether using a balanced, low-chloride fluid instead of 0.9% saline is safe and improves kidney function after deceased donor kidney transplantation is unknown.

**Methods:**

BEST-Fluids is an investigator-initiated, pragmatic, registry-based, multi-center, double-blind, randomized controlled trial. The primary objective is to compare the effect of intravenous Plasma-Lyte 148 (Plasmalyte), a balanced, low-chloride solution, with the effect of 0.9% saline on the incidence of delayed graft function in deceased donor kidney transplant recipients. From January 2018 onwards, 800 participants admitted for deceased donor kidney transplantation will be recruited over 3 years in Australia and New Zealand. Participants are randomized 1:1 to either intravenous Plasmalyte or 0.9% saline peri-operatively and until 48 h post-transplant, or until fluid is no longer required; whichever comes first. Follow up is for 1 year. The primary outcome is the incidence of delayed graft function, defined as dialysis in the first 7 days post-transplant. Secondary outcomes include early kidney transplant function (composite of dialysis duration and rate of improvement in graft function when dialysis is not required), hyperkalemia, mortality, graft survival, graft function, quality of life, healthcare resource use, and cost-effectiveness. Participants are enrolled, randomized, and followed up using the Australia and New Zealand Dialysis and Transplant (ANZDATA) Registry.

**Discussion:**

If using Plasmalyte instead of 0.9% saline is effective at reducing delayed graft function and improves other clinical outcomes in deceased donor kidney transplantation, this simple, inexpensive change to using a balanced low-chloride intravenous fluid at the time of transplantation could be easily implemented in the vast majority of transplant settings worldwide.

**Trial registration:**

Australian New Zealand Clinical Trials Registry: ACTRN12617000358347. Registered on 8 March 2017. ClinicalTrials.gov: NCT03829488. Registered on 4 February 2019.

## Administrative information

Note: the numbers in curly brackets in this protocol refer to Standard Protocol Items: Recommendation for Interventional Trials (SPIRIT) checklist item numbers. The order of the items has been modified to group similar items (see http://www.equator-network.org/reporting-guidelines/spirit-2013-statement-defining-standard-protocol-items-for-clinical-trials/).

**Table:** 

**Title {1}**	Study Protocol for Better Evidence for Selecting Transplant Fluids (BEST-Fluids): a pragmatic, registry-based, multi-center, double-blind, randomized controlled trial evaluating the effect of intravenous fluid therapy with Plasma-Lyte 148 versus 0.9% saline on delayed graft function in deceased donor kidney transplantation.
**Trial registration {2a and 2b}**	Australian New Zealand Clinical Trials Registry ACTRN12617000358347 ClinicalTrials.gov identifier NCT03829488
**Protocol version {3}**	Version 3.1, 2 March 2020
**Funding {4}**	1. Medical Research Future Fund (MRFF) *Rare Cancers, Rare Diseases and Unmet Needs* Grant 2018 (APP1152390) 2. Health Research Council of New Zealand Project Grant 2017 (17/414) 3. Better Evidence And Translation in Chronic Kidney Disease (BEAT-CKD) grants 2016 and 2017 (from NHMRC program grant 2014 APP1092957, Chief Investigator Jonathan Craig) 4. Royal Australasian College of Physicians *Jacquot Research Establishment Fellowship* grants 2017 and 2018 (awarded to Dr. Michael Collins) 5. Baxter Investigator Initiated Research Grant (Medication Delivery) 2017 (in kind support; provision of trial fluids)
**Author details {5a}**	1. Department of Renal Medicine, Auckland City Hospital, Auckland District Health Board, Auckland, New Zealand 2. Department of Medicine, Faculty of Medical and Health Sciences, University of Auckland, Auckland, New Zealand 3. Australasian Kidney Trials Network, The University of Queensland, Brisbane, Australia 4. Department of Nephrology, Princess Alexandra Hospital, Brisbane, Australia 5. Australia and New Zealand Dialysis and Transplant (ANZDATA) Registry, South Australian Health and Medical Research Institute (SAHMRI), Adelaide, Australia 6. Department of Medicine, The University of Adelaide, Adelaide, Australia 7. Central and Northern Adelaide Renal and Transplantation Service, Royal Adelaide Hospital, Adelaide, Australia 8. Sydney School of Public Health, Faculty of Medicine and Health, The University of Sydney, Sydney, Australia 9. Department of Critical Care Medicine, Auckland City Hospital, Auckland, New Zealand 10. Department of Nephrology, Austin Health, Melbourne, Australia 11. Department of Medicine (Austin), The University of Melbourne, Parkville, Melbourne, Australia 12. Department of Anaesthesia, Austin Health, Melbourne, Australia 13. The University of Melbourne, Department of Surgery, Austin Health, Melbourne, Australia 14. Department of Renal Medicine, Royal Prince Alfred Hospital, Sydney, Australia 15. Charles Perkins Centre, The University of Sydney, Sydney, Australia * **Correspondence:** Dr. Michael G. Collins, FRACP, PhD, Department of Renal Medicine, Auckland District Health Board, Private Bag 92,024, Auckland 1142, New Zealand Email: michael.collins@adhb.govt.nz
**Name and contact information for the trial sponsor {5b}**	The University of Queensland acting through Australasian Kidney Trials Network (AKTN) Email: aktn@uq.edu.au
**Role of sponsor {5c}**	The sponsor is the coordinating center for the trial and is involved in overall study activities including study design, collection, management, analysis and interpretation of data, writing of the report, and decision to submit the report for publication. Baxter Healthcare will be provided with the report for review. Neither Baxter Healthcare nor the other funders have any involvement in the design, oversight, data collection, analysis, interpretation, or reporting of the study, or the decision to submit the report for publication.

## Introduction

### Background and rationale {6a}

End-stage kidney disease (ESKD) is a significant public health problem worldwide and its treatment imposes a high healthcare burden and cost. Kidney transplantation is considered the best treatment for ESKD, offering improved survival and quality of life at significantly lower cost than dialysis [1, 2]. However, there is a shortage of available donor organs, and many kidney transplants fail prematurely due in part to injury sustained around the time of transplantation.

Delayed graft function (DGF), the requirement for dialysis due to poor kidney function in the early post-operative period, is an increasingly frequent complication of deceased donor kidney transplantation, with a current estimated incidence of 30% or greater [3, 4]. In addition to a longer length of hospital stay, higher costs, and an increased risk of acute rejection in the immediate post-transplant period [5–7], DGF is associated with reduced graft function and inferior graft and patient survival in the long term [6, 8].

DGF reflects acute kidney injury caused by ischemia-reperfusion injury during transplantation, and is driven by donor, recipient, and transplant factors [9, 10]. It results from hypoxia and inflammation induced by hypotension, vasospasm, cytokine release and activation of the innate immune system [10], and it can occur prior to and during organ procurement, during storage and transport, and in the recipient following reperfusion of the graft. Interventions that have reduced the incidence of DGF include treatment of the deceased organ donor with hypothermia or dopamine prior to retrieval [11, 12] and machine perfusion of the donor kidney during transport and storage [13]. However, logistics and high costs remain significant barriers to the widespread application of these approaches, and there are currently no established interventions in the recipient that can reduce the incidence of DGF.

Intravenous fluid therapy is a critical, albeit inexpensive, aspect of peri-operative care for the transplant recipient that is required to maintain intravascular volume and optimize graft perfusion and function. Isotonic sodium chloride (“normal” or 0.9% saline) is the standard crystalloid solution utilized at most centers [14]. However, 0.9% saline may be harmful due to its high chloride concentration relative to plasma (Table 1), which causes hyperchloremic metabolic acidosis. This in turn may lead to acute kidney injury and DGF as a result of renal vasoconstriction and kidney tissue edema [15–17]. Furthermore, metabolic acidosis frequently leads to hyperkalemia, increasing the risk of arrhythmias and cardiac instability, further exacerbating kidney injury [18]. Therefore, utilizing a balanced low-chloride crystalloid solution, such as Plasma-Lyte 148 (Plasmalyte) as an alternative to 0.9% saline may result in improved outcomes after kidney transplantation.

**Table 1:** Physicochemical characteristics of selected electrolyte solutions and human plasma

	**0.9% Saline**	**Ringers Lactate**	**Plasmalyte**	**Human plasma**
Sodium (mmol/L)	154	130	140	136–145
Potassium (mmol/L)	0	4	5	3.5–5.0
Magnesium (mmol/L)	0	0	1.5	0.8–1.0
Calcium (mmol/L)	0	2.7	0	2.2–2.6
Chloride (mmol/L)	154	109	98	98–106
Acetate (mmol/L)	0	0	27	0
Gluconate (mmol/L)	0	0	23	0
Lactate (mmol/L)	0	28	0	0
pH	6.8	6.5	7.4	7.35–7.45
Osmolarity (mOsm/L)	308	273	295	280–296
Calculated osmolality in vivo^a^ (mOsm/kg)	287	254	271	
Isotonic with plasma	Yes	No^b^	Yes	

^a^Sodium and chloride are only partially osmotically active with an osmotic coefficient of 0.926

^b^Ringers Lactate is hypotonic compared with plasma

Studies in surgical and intensive care patients administered relatively low volumes of fluid have shown modest reductions in measures of acute kidney injury and dialysis, with balanced low-chloride crystalloids compared with 0.9% saline [19–23]. In kidney transplantation, studies have shown reduced acidosis with low-chloride solutions compared with 0.9% saline, however, they have been insufficiently powered to detect important differences between the groups for DGF and other transplant outcomes [24]. Moreover, these results are not necessarily generalizable to the typical deceased donor kidney transplant setting, as two thirds of studies involved recipients of living donor kidneys (at very low risk of DGF), and trial fluids were only given intra-operatively in relatively low volumes. However, in a recent blinded randomized trial conducted in deceased donor kidney transplant recipients who received fluid volumes more typical of clinical practice (median 6–7 L from surgery until post-operative day two), the incidence of hyperkalemia was significantly lower with Plasmalyte compared with 0.9% saline, and a post-hoc analysis showed improvements in several measures of graft function [25]. Definitive trials in high-risk populations, including deceased donor kidney transplant recipients, are therefore still required.

The Better Evidence for Selecting Transplant Fluids (BEST-Fluids) trial is a randomized controlled trial designed to test the hypothesis that intravenous (IV) fluid therapy with a balanced low-chloride crystalloid solution, Plasmalyte, compared with 0.9% saline, will reduce the incidence and severity of acute kidney injury and delayed graft function in deceased donor kidney transplant recipients.

### Objectives {7}

The primary objective of the BEST-Fluids trial is to compare the effect of peri-operative IV fluid therapy using Plasmalyte with that of fluid therapy using 0.9% saline on the incidence of delayed graft function (DGF) in deceased donor kidney transplant recipients.

The secondary objectives are to determine whether peri-operative IV therapy using Plasmalyte, compared with 0.9% saline, (1) improves the recovery of graft function in the first week after transplantation; (2) reduces the number of dialysis treatments required and the duration of dialysis dependence after transplantation; (3) reduces the incidence and severity of hyperkalemia; (4) improves graft survival and death-censored graft survival at 12 months; (5) improves graft function up to 12 months post-transplant; (6) improves health-related quality of life; (7) reduces hospital length of stay and health-related costs; and (8) is cost-effective.

### Trial design {8}

**Fig. 1:**
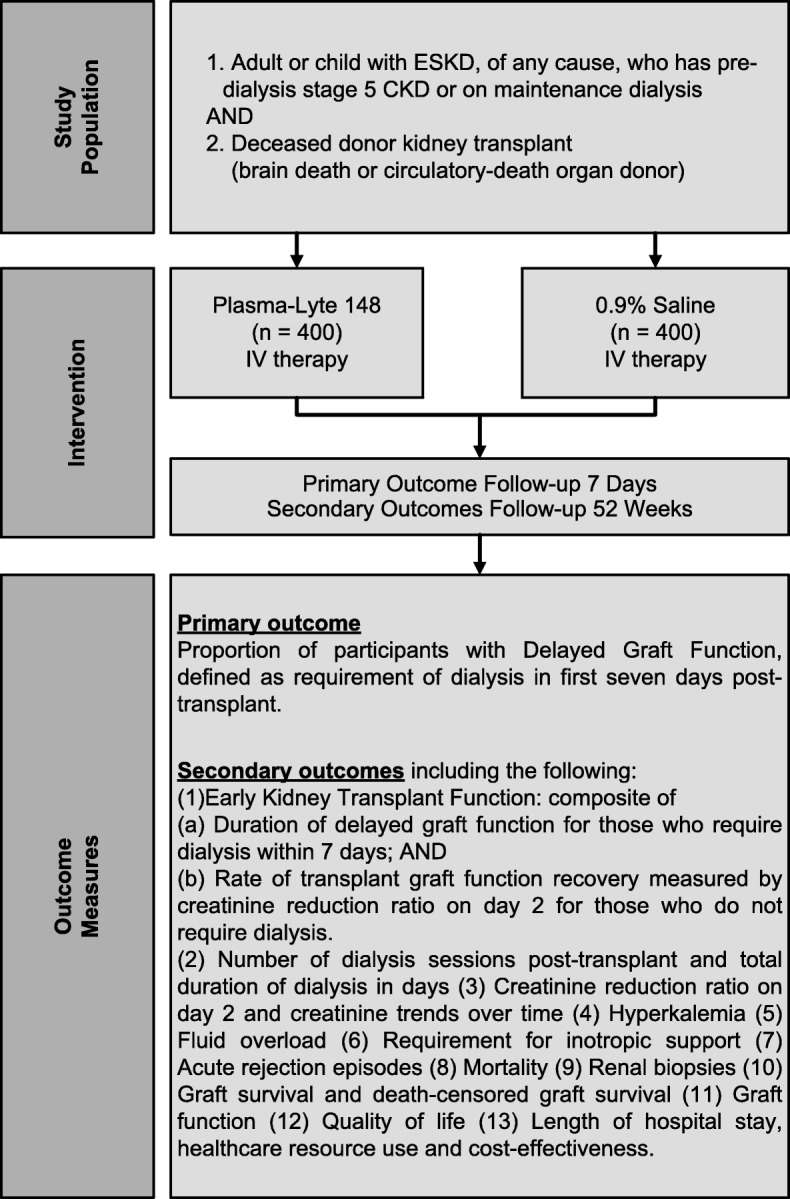
Outline of the BEST-Fluids trial. ESKD, end-stage kidney disease; CKD, chronic kidney disease

**Fig. 2:**
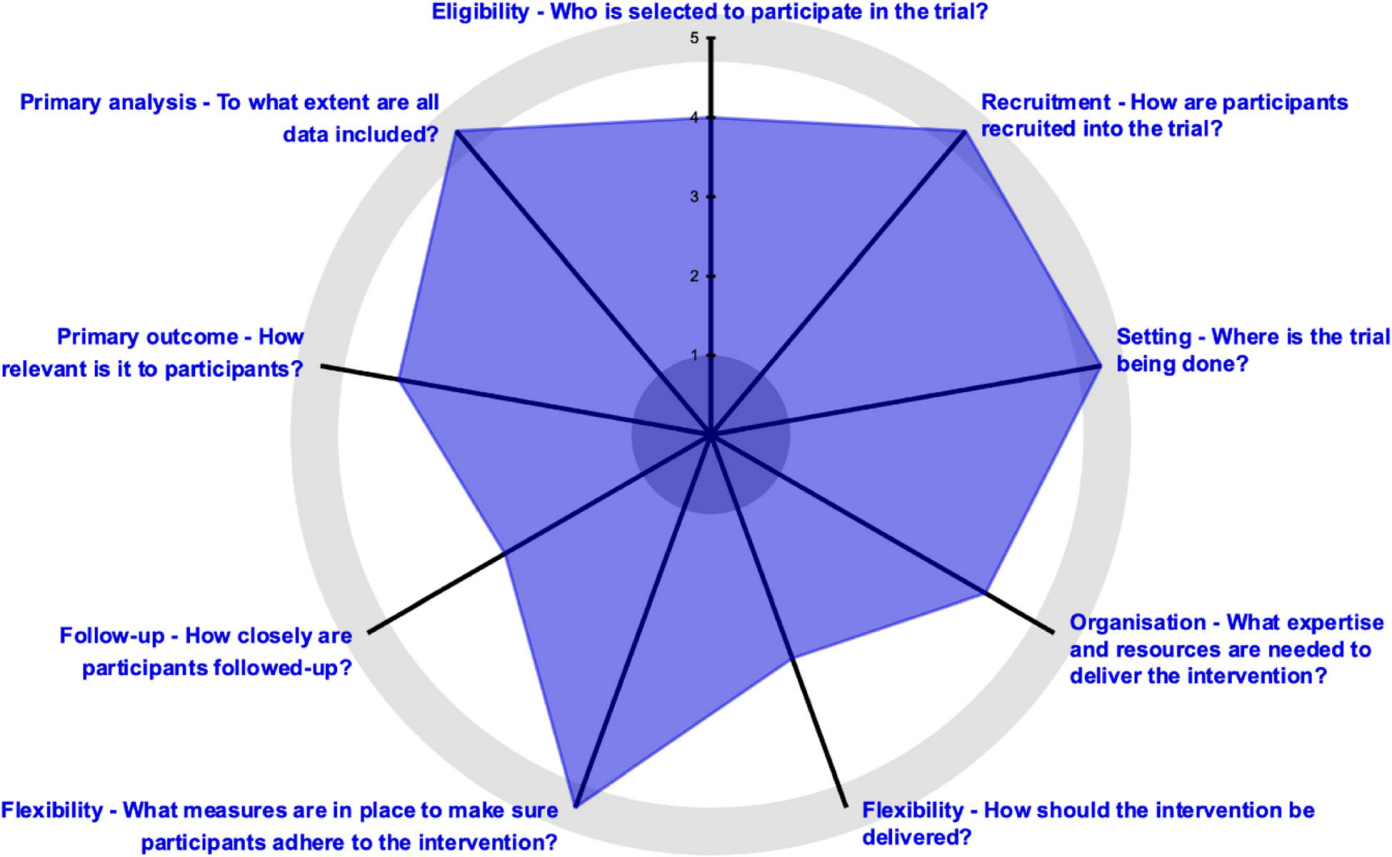
Pragmatic aspects of trial design. The Pragmatic-Explanatory Continuum Indicator Summary 2 (PRECIS-2) wheel for the BEST-Fluids trial. Scores in each domain range from 1 (very explanatory) to 5 (very pragmatic) as described in Loudon et al. [26]. This PRECIS-2 wheel was generated using the tool available at www.precis-2.org

BEST-Fluids is an investigator-initiated, pragmatic, registry-based, prospective, multi-center, parallel-group, double-blind, randomized, active-controlled, superiority trial. An outline of the trial is shown in Fig. 1. An assessment of the pragmatic design aspects of the trial using the Pragmatic-Explanatory Continuum Indicator Summary 2 (PRECIS-2) tool [26] is shown in Fig. 2 and described in more detail in Additional file 1.

The Australasian Kidney Trials Network (AKTN, The University of Queensland) is the sponsor and coordinating center for the trial. The Australia and New Zealand Dialysis and Transplant (ANZDATA) Registry is being used for participant enrolment, randomization, and follow-up data collection.

The first trial participant was enrolled on 30 January 2018 and recruitment is expected to be completed in December 2020, with 12-month follow up of all participants scheduled to be completed by December 2021.

## Methods: participants, intervention, and outcomes

### Study setting {9}

A total of 800 participants undergoing deceased donor kidney transplantation will be recruited at hospitals in Australia and New Zealand. Additional file 2 contains a list of the study sites.

### Eligibility criteria {10}

#### Inclusion criteria

The inclusion criteria are:

1. Adult or child with end-stage kidney disease from any cause and on maintenance dialysis or who has chronic kidney disease with an estimated glomerular filtration rate (eFGR) of < 15 mL/min/1.73m^2^ (as determined by the Chronic Kidney Disease - Epidemiology Collaboration (CKD-EPI) equation for adults [27] or the bedside Schwartz equation for children) [28];
2. Planned deceased donor kidney transplant from an organ donor in whom brain death (DBD) or circulatory death (DCD) is expected to occur within the next 24 h;
3. Written informed consent or consent given by their parent or guardian (if the participant is age < 18 years) or other authorized person.

#### Exclusion criteria

The exclusion criteria are:

1. Planned live donor kidney transplant (except where this is cancelled in favor of transplantation from a deceased donor);
2. Planned multi-organ transplant (dual or *en-bloc* kidney transplants are not excluded);
3. A child with weight < 20 kg or a child who the treating physician believes should not be included in a blinded study of fluids due to their small body size;
4. Known hypersensitivity to the trial fluid preparations or packaging.

The risk of DGF after live donor kidney transplantation [10] is very low (< 10%), thus patients undergoing this procedure are excluded. Multi-organ (heart-kidney, liver-kidney or pancreas-kidney) transplants have different surgical and management approaches that would confound the trial. Small children, particularly those under 20 kg in weight, are more susceptible to electrolyte imbalances due to the volumes of IV fluid therapy required post-transplantation, and they have been excluded for safety reasons. Patients with known hypersensitivity to the solutions or trial fluid packaging are also excluded for safety reasons.

### Who will take informed consent? {26a}

Patients who are admitted for a deceased donor kidney transplant at participating hospitals are invited to participate in the trial. Clinical staff (physicians or resident medical staff) determine participant eligibility, discuss the trial, and seek informed consent during routine transplant pre-operative assessments. All clinical staff involved in obtaining consent and enrolling participants have received specific training in the trial and the requirements of Good Clinical Practice (GCP); they do not necessarily need to be investigators or research staff.

### Additional consent provisions for collection and use of participant data and biological specimens {26b}

Additional written consent is sought from Australian participants to utilize routinely collected individual health data on outpatient healthcare use from the Medicare Benefits Schedule (MBS) and prescription medicines from the Pharmaceutical Benefits Scheme (PBS), which will be used in the economic evaluation. This additional consent is not required in New Zealand as specific consent to utilize health information linked to the National Health Index database is incorporated into the main consent form.

Participants are also asked for their consent to have their radiology images, data, and reports from ultrasound scans or nuclear medicine scans, performed post-transplant, collected for analysis in a separate imaging sub-study. No additional biological samples outside those collected as part of routine clinical care are being collected from participants.

## Interventions

### Explanation for the choice of comparators {6b}

There is physiological and clinical evidence to support the hypothesis that using a balanced low-chloride solution, such as Plasmalyte, as IV therapy in deceased donor kidney transplantation may reduce the potential for adverse effects, including acute kidney injury and DGF, which could occur due to the high-chloride content of 0.9% saline. Available low-chloride solutions that could be utilized as IV crystalloid therapy in transplantation include Plasmalyte, Hartmanns or Ringers Lactate, and Elomel Isoton (Fresenius Kabi). Hartmanns/Ringers Lactate has been used intra-operatively in trials involving transplant recipients, but has the disadvantage of being a hypotonic solution (osmolality 254 mOsm/kg), increasing the risk of post-operative hyponatremia, especially when used in significant volumes as occurs in transplantation. Elomel Isoton, which has been used in a trial of deceased donor transplant recipients, is similar to Plasmalyte, except that acetate is the sole buffer (Plasmalyte contains both acetate and gluconate buffers); however, it is not currently licensed in Australia and New Zealand. Plasmalyte is licensed in Australia, New Zealand, and many other countries, is readily available and already in use at many centers, and was recently evaluated for safety and potential efficacy in a small trial involving deceased donor kidney transplant recipients [29]. Thus, Plasmalyte was selected as the experimental low-chloride crystalloid fluid therapy for this trial.

Numerous observational studies in the surgical setting have associated the use of 0.9% saline with an increased risk of acute kidney injury [19, 22, 23, 30–33]. However, as with all observational studies, these findings may be affected by selection bias and residual confounding, making causal inferences problematic. Two prospective pilot studies (the SPLIT-ICU and SALT trials) in the intensive care setting [34, 35] evaluating the effects of buffered crystalloid solutions versus 0.9% saline reported no differences between patient groups in the rate of major adverse kidney events. More recently, in two large multiple crossover cluster trials involving > 20,000 non-critically ill and critically ill adult patients, the use of Hartmann’s solution or Plasmalyte resulted in a lower rate of major adverse kidney events when compared to 0.9% saline [20, 21]. Importantly, these studies did not discriminate between patients undergoing kidney transplant and other patients and did not involve individual blinding of the treatment assignments.

In the context of major surgery, the effects of restricting peri-operative use of IV chloride on kidney injury were examined in the LICRA and SOLAR trials [36, 37]. In both studies, there was no clinically meaningful difference in the risk of a composite of in-hospital mortality and major post-operative complications, including acute kidney injury. Patients undergoing kidney transplantation were excluded. Accordingly, there are no current guidelines on which crystalloid solution should be used in deceased donor kidney transplantation.

Currently, IV therapy with 0.9% saline is the standard of care at the majority of transplant centers in Australia and New Zealand. In a 2016 survey of 22 renal transplant centers conducted by several of the authors (MC, MF, DR, CH, and SC), 64% reported using 0.9% saline intra-operatively and 87% reported using it post-operatively (unpublished observations), which is similar to published studies from other countries [14]. Hence, 0.9% saline was chosen as the standard of care to use as the control therapy in this trial.

### Intervention description {11a}

Participants will be randomized to one of two blinded fluid-therapy groups:

1. Plasmalyte: low-chloride, balanced crystalloid solution, Plasma-Lyte 148 (approx. pH 7.4) IV Infusion (Baxter Healthcare, Toongabbie, NSW, Australia) or
2. 0.9% Saline: isotonic Sodium Chloride (0.9%) IV Infusion BP (Baxter Healthcare, Toongabbie, NSW, Australia).

The allocated trial fluid is administered blinded as the routine IV fluid therapy for all maintenance, replacement, and resuscitation purposes. The fluid is administered from randomization onwards pre-operatively, intra-operatively, and post-operatively, until either IV fluid therapy is no longer required or until 48 h post-transplant, whichever is earliest. The rate and volume of fluid administration are not mandated by the study protocol and are prescribed by treating physicians according to usual center practice.

Participants are each allocated an individual box containing 12 × 1000 mL bags of blinded trial fluid labelled with a unique 4-digit study code (treatment pack number). The treatment pack number is provided by the randomization system (accessed via ANZDATA), corresponding with an available box at the study site. The box of allocated fluids remains physically with the participant during their hospital stay, and is transported with them to the operating theatre and other hospital locations as required. A further two boxes of 12 × 1000 mL fluid bags can be allocated by the randomization system prior to 48 h post-transplant, allowing up to 36 L of trial fluid to be used per participant if required. Each uniquely labelled box can be allocated to one participant only.

### Criteria for discontinuing or modifying allocated intervention {11b}

Blinded trial fluids may be temporarily or permanently discontinued by the treating clinician or investigator at any time if (1) the IV fluids are no longer indicated or required, (2) there is an indication for a specific open-label fluid that cannot be co-administered with study fluids, (3) a serious adverse event occurs that may be related to fluid therapy, (4) continuation is deemed not to be in the participant’s best interests, or (5) if requested by the treating physician or participant.

Once the 48-h post-transplant timepoint is reached, participants who continue to require IV fluids may be continued on trial fluids beyond 48 h, until the stock of fluid bags within a previously allocated box is exhausted. Alternatively, participants may be converted to open-label fluids prescribed by the treating physician at their discretion.

### Strategies to improve adherence to interventions {11c}

A face-to-face initiation meeting was held prior to the commencement of the trial at each site. Site investigators and research staff engaged with their multidisciplinary teams and provided training and tailored local resources to support adherence to the intervention, including checklists, trial alert notices in the medical records, handover documents affixed to the patient’s hospital bed, and trial fluid accountability logs. Presentations on the trial were typically given by site investigators at meetings of clinicians involved in transplant peri-operative care, including transplant and anesthesia departments, to maximize awareness and engagement. Monitoring visits by the staff from the trial coordinating center are conducted periodically to evaluate risk mitigation strategies and confirm adequate study fluid supplies, training, and resources to meet site needs.

### Relevant concomitant care permitted or prohibited during the trial {11d}

The use of other non-trial fluids, such as blood products, are permitted as per routine clinical care and treating physician discretion. The use of non-trial open-label crystalloids is permitted in specific circumstances (e.g. hypotonic solutions for hypernatremia, or for administration of IV medications), but is otherwise discouraged in the absence of a compelling clinical indication.

All participants receive usual transplant management as per the local standard of care. This includes peri-operative anesthesia and invasive monitoring, circulatory support, surgical care, immunosuppression, routine prophylactic measures, the management of fluid overload, hyperkalemia and other electrolyte disturbances, and other complications. Hyperkalemia is treated as per standard practice and local protocols. Dialysis is performed for standard clinical indications, as determined by treating clinicians. Further details of protocol recommendations for concurrent participant management, including the use of open-label crystalloids and other fluid therapy, are outlined in Additional file 3.

### Provisions for post-trial care {30}

Following completion of the trial intervention at 48 h post-transplant, participants are followed up until 12 months post-transplant. All post-transplant care is provided by the hospital where the transplant takes place or other local hospital, as per routine practice. The Sponsor has indemnity insurance to cover those who suffer from potential harm due to participation in the trial. New Zealand participants are eligible to apply for compensation from the New Zealand Accident Compensation Corporation (ACC) for treatment-related injuries that occur due to participation in the trial.

### Outcomes {12}

#### Primary outcome measure

The primary outcome is the proportion of participants with DGF, defined as those receiving treatment with any form of dialysis in the first 7 days after transplant.

#### Secondary outcome measures

The secondary outcome measures include the following:

1. Early Kidney Transplant Function, measured using a ranked composite of (a) the duration of delayed graft function in days for the participants requiring dialysis, and (b) the rate of transplant graft function recovery measured by creatinine reduction ratio on day 2 (CRR2) [38] for the participants who do not receive dialysis. CRR2 (%) = ([creatinine_day 1_-creatinine_day 2_]*100)/creatinine_day1_ (see Additional file 4 for further details).
2. The number of dialysis sessions (in the first 28 days), and the total duration of dialysis in days (from transplant to the final dialysis treatment).
3. Creatinine reduction ratio on day 2 post-transplant, and the proportion of participants with a decrease in serum creatinine of ≥ 10% on 3 days consecutively in the first 7 days post-transplant.
4. Serum creatinine trends over 52 weeks.
5. Incidence of serum potassium ≥ 5.5 mmol/L and peak potassium level in the first 48 h post-transplant.
6. Treatment for hyperkalemia with dialysis, IV calcium, insulin, β-agonists, sodium bicarbonate, or ion exchange resins in the first 48 h post-transplant.
7. Incidence of significant fluid overload, defined as > 5% weight gain (baseline to day 2).
8. Aggregate urine output until day 2 post-transplant.
9. Requirement for inotropic support, both intra-operatively and post-operatively, to day 2.
10. Number of acute rejection episodes in the first 52 weeks.
11. Number of renal transplant biopsies performed in the first 28 days post-transplant.
12. Mortality up to 52 weeks.
13. Graft survival and death-censored graft survival at 52 weeks.
14. Graft function (eGFR derived from serum creatinine using the CKD-EPI equation [27] (adults) or the bedside Schwartz equation [28] (children) at 4, 12, 26, and 52 weeks.
15. Health-related quality of life, as measured by the EuroQol five dimensions questionnaire (EQ-5D-5 L) for adults and by the EuroQol five dimensions Youth questionnaire (EQ-5D-Y) for children aged < 18 years.
16. Length of hospital stay, healthcare resource use and cost-effectiveness over 12 months.

### Participant timeline {13}

**Fig. 3:**
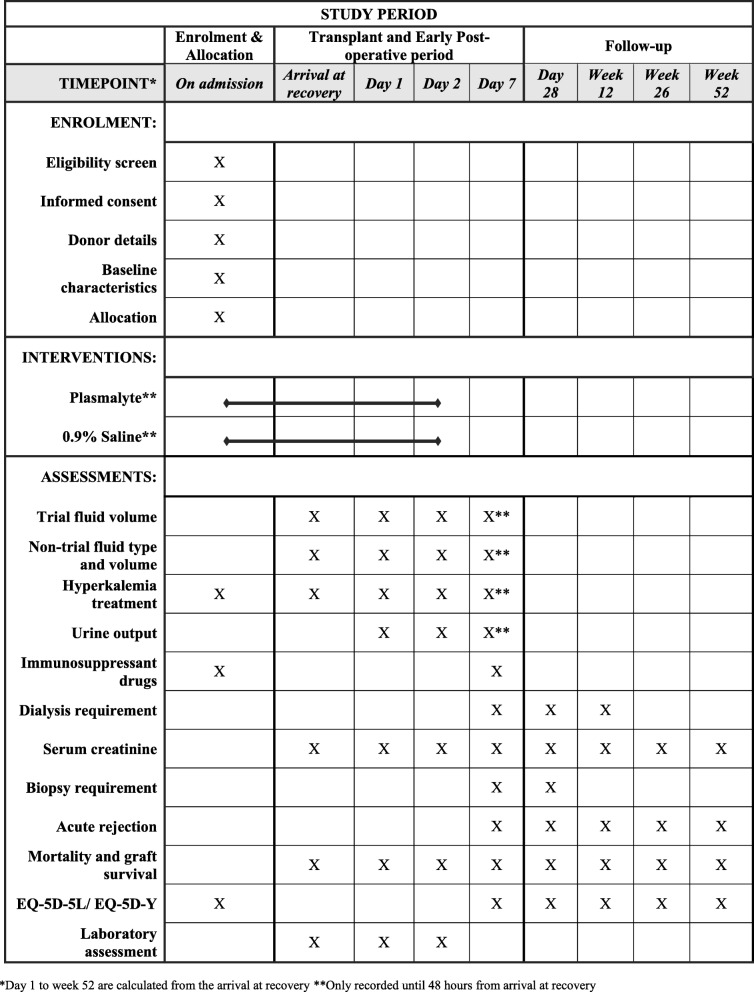
Participant timeline. Standard Protocol Items: Recommendations for Interventional Trials (SPIRIT) checklist. Enrolment, interventions and assessments. EQ-5D-5L, EuroQol five dimensions, 5 levels questionnaire; EQ-5D-Y, EuroQol five dimensions, Youth questionnaire

Participants are followed up in the study from randomization until 52 weeks after transplant surgery. Figure 3 shows the schedule of enrolment, interventions, and assessments. Data collection occurs at baseline, post-operatively (on arrival to the post-anesthetic recovery unit after transplant surgery), on days 1, 2, 7, and 28, and on weeks 12, 26, and 52 (see Additional file 5 for further details).

### Sample size {14}

The sample size for the BEST-Fluids trial (800 participants) is based on a comparison between two independent groups of the proportions of participants experiencing the primary outcome measure of DGF. The effect size, or minimum clinically important difference, was determined by considering that a relative risk (RR) reduction of approximately 25% (RR of 0.75) for the incidence of the primary outcome would be both clinically meaningful and within the range of biological plausibility for the association between DGF and Plasmalyte versus 0.9% saline. The latter is based on trends observed in (1) the Weinberg trial [29]: RR of dialysis within 48 h post-transplant of 0.78 (95% confidence interval (CI) 0.47 to 1.28) and (2) a recently published before-and-after non-randomized interventional study [39]: RR for dialysis within 48 h of 0.3 (95% CI 0.10 to 0.97; adjusted odds ratio 0.14; 95% CI 0.03 to 0.48), as the most current and relevant data available.

A sample size of 722 participants (361 per group) will have 80% power at a 5% two-sided significance level to show an estimated absolute difference between the groups of 10% (41% versus 31%), with an estimated overall incidence of DGF of 36% and RR for Plasmalyte versus 0.9% saline of 0.76. Allowing for 4.0% non-adherence (estimated 2% drop-out from the Plasmalyte group and 2% drop-in) and up to 1% loss to follow up for the primary outcome measure (e.g. due to death or withdrawal of consent within 7 days), an adjusted sample size of 792 participants is required. To allow for fluctuations in these estimates, a total of 800 participants will be recruited.

While the target recruitment allows for 1% loss to follow up, it is expected that loss to follow-up in this study for both the primary outcome (7 days) and secondary outcomes (up to 12 months) will be close to zero due to (1) the very close clinical follow up that renal transplant recipients routinely receive, (2) the short timeframe for ascertainment of the primary outcome (7 days post-transplant), and (3) the fact that transplant recipients are highly motivated patients who typically maintain strong engagement with their care providers.

### Recruitment {15}

BEST-Fluids is being conducted at 13 adult hospitals and 3 children’s hospitals that perform kidney transplants in Australia and New Zealand. Collectively these hospitals performed more than 500 deceased donor kidney transplants annually in the year prior to trial commencement; thus, approximately 50–55% of eligible patients overall need to be enrolled to meet the target sample size of 800 over 3 years.

Strategies have been implemented to achieve optimal participant enrolment: comprehensive site engagement with members of the multi-disciplinary clinical team prior to initiation of the trial, a pragmatic trial design with broad eligibility criteria, recruitment of participants by clinical staff who usually undertake the pre-operative assessments prior to transplant at each site, consent forms written in plain English (with interpreters available where required), a simple, rapid randomization process accessed online using ANZDATA, and the alignment of trial processes and the study intervention with usual clinical care. Each site has been provided with training on and resources for the trial, including short online randomization training videos to familiarize them with the ANZDATA Registry web-based platform used for enrolment. With few exceptions, participants are already being treated with dialysis and are registered in the ANZDATA Registry prior to being admitted for transplant, which greatly simplifies the process of enrolment. On-call 24-h telephone support is provided to clinical staff who are enrolling participants, with the ability to access assistance for enrolment and randomization from an investigator on the coordinating committee.

## Assignment of interventions: allocation

### Sequence generation {16a}

Participants are randomized 1:1 to either Plasmalyte or 0.9% saline, using an adaptive minimization method [40]. The minimization algorithm is designed to ensure balance across the two treatment groups for factors associated with DGF and graft outcomes, including:

1. Transplant center [41];
2. Deceased donor status (donation after brain death, donation after circulatory death);
3. Machine perfusion (no, yes); and
4. Australian Kidney Donor Risk Index (KDRI) tertile. The KDRI is a composite measure of donor quality based on eight donor characteristics known at the time of transplantation [42, 43].

### Concealment mechanism {16b}

The list of unique study codes (treatment pack numbers) and treatment allocations accessed by the minimization algorithm, and the algorithm itself, are stored centrally in an external web-based randomization system provided by the NHMRC Clinical Trials Centre at The University of Sydney, and are not accessible to investigators, study staff, or staff at the ANZDATA Registry.

### Implementation {16c}

The code for generating the treatment pack numbers and treatment allocations was developed by an un-blinded statistician who is not a member of the study team. Participants are enrolled and randomized by site staff using the ANZDATA Registry web-based platform, which communicates with the randomization system via a secure web-based connection built into the registry system that is not accessible to end users. The randomization system returns a unique participant study identification number and treatment pack number to the ANZDATA Registry, which then displays this information to the site staff member and sends an auto-email to their email address and the AKTN trial coordinating center.

## Assignment of interventions: blinding

### Who will be blinded? {17a}

Study participants, treating physicians and other healthcare providers, outcome assessors, research staff, study investigators, staff at the AKTN trial coordinating center, ANZDATA Registry staff, and the primary trial statistician are blinded. Details of the treatment allocations and unique treatment pack numbers are known only to a specified un-blinded statistician, the Baxter Healthcare manufacturing team, and data staff at the NHMRC Clinical Trials Centre.

**Fig. 4:**
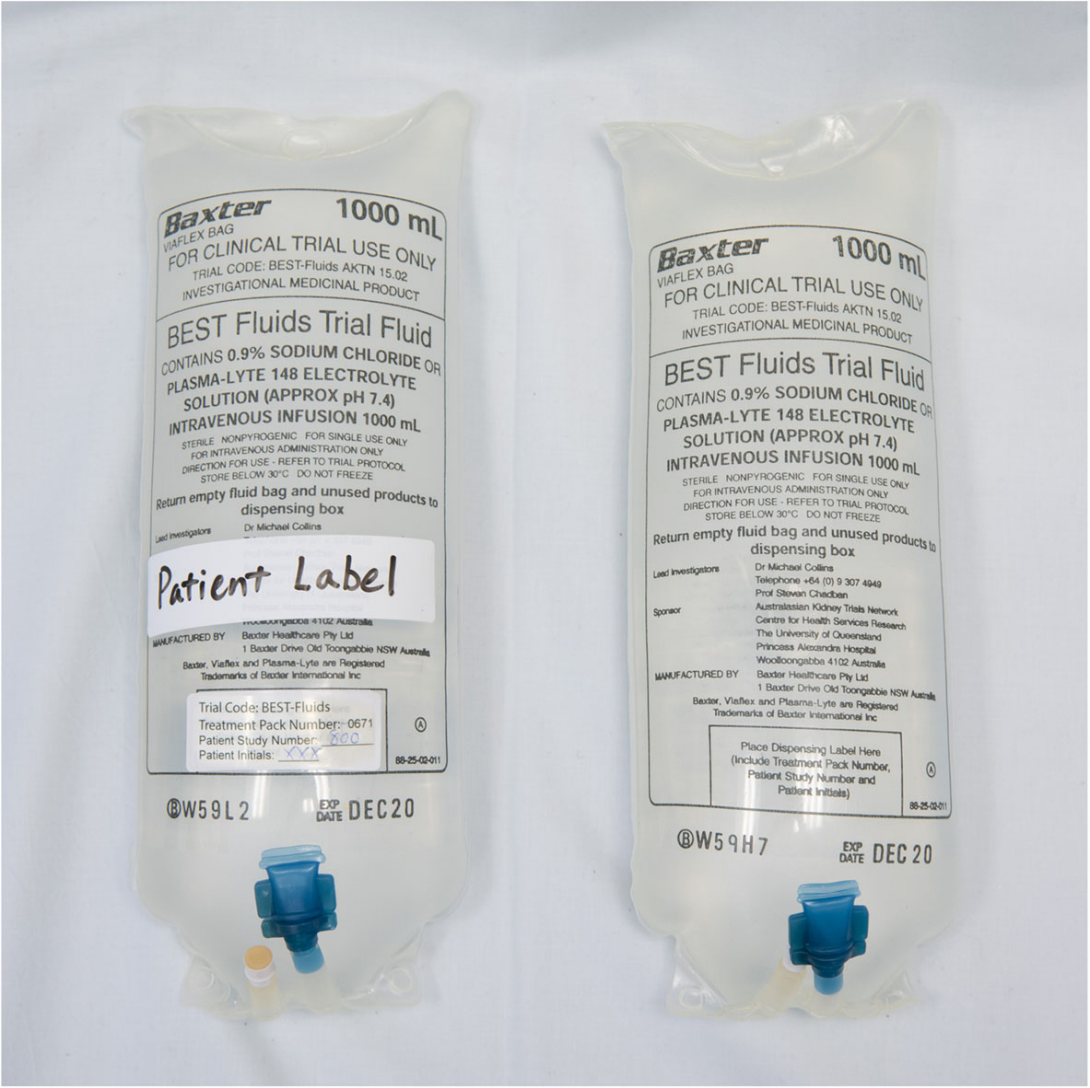
Masking of study fluid. The printed label design on the study fluid bag does not allow identification of the type of study fluid (right hand image). Two adhesive stickers with the unique study code (treatment pack number) are attached to the exterior protective over-pouch (not shown), one of which is applied to the bag itself, along with a patient label, when the bag is opened by clinical staff (left hand image)

The Plasmalyte and 0.9% saline preparations and packaging used in this study are manufactured by Baxter Healthcare Pty Ltd. (Toongabbie, NSW, Australia) specifically for the trial, and, apart from their labelling, are identical to those used in standard clinical practice in Australia and New Zealand. The trial fluid allocation is masked by using a specifically designed printed trial label that does not allow the identification of the specific fluid type (Fig. 4). Prior to shipment of the boxes from the manufacturer to study sites, each trial fluid box is labelled externally with the 4-digit treatment pack number. A set of two pre-printed adhesive labels with the same code are affixed to the outside of the protective covers over individual bags in the box, to allow the bags within the box to be labelled by clinical staff once opened.

### Procedure of unblinding if needed {17b}

Unblinding is not permitted during the trial except in the event of a serious adverse event. One of the lead Principal Investigators (MC) is the main contact for sites to discuss any request for unblinding and will remain blinded. If unblinding is deemed necessary, the time, date and reason will be recorded and the un-blinded statistician will contact the healthcare professional involved at the site. Only those who need to know will be made aware of the treatment allocation. The participant will remain in the study and, where possible, continue their allocated study treatment.

## Data collection and management

### Plans for assessment and collection of outcomes {18a}

Participants are enrolled, randomized and followed up using ANZDATA, which is a long-standing, bi-national clinical quality registry that collects incidence, prevalence, and outcome data on dialysis and kidney transplantation in Australia and New Zealand. Trial processes are embedded within the registry to improve trial efficiency by avoiding data entry duplication, enhancing integration into routine care, and facilitating long-term follow up. Clinical and laboratory data are collected by study staff from the participant’s medical record. Information on health-related quality of life is collected by participant completion of the EQ-5D-5L questionnaire for adults, and the EQ-5D-Y questionnaire for children.

### Plans to promote participant retention and complete follow up {18b}

It is anticipated that participants will be in hospital for all visits prior to day 7, with subsequent visits timed to coincide with routine outpatient clinic appointments undertaken as part of standard post-transplant care. The study team collect the data required for each visit from clinical records of outpatient clinic attendances and laboratory tests performed as part of routine care at the location this is being provided. If a participant transfers to another center/referring renal unit away from the primary transplant center for ongoing care, the study visit data can be collected from day 28 (visit 6) onwards via telephone or electronic mail from local treating physicians/nursing staff, and via post, email or telephone from participants for the collection of the EQ-5D quality of life questionnaires. Participants are not required to attend the study site for any study visits after hospital discharge. If a participant no longer wishes to complete the quality of life questionnaires, other study data will continue to be collected from clinical records and the ANZDATA Registry, unless the participant specifically withdraws consent for this to occur.

### Data management {19}

The ANZDATA Registry web-based system is used for collection of trial outcomes data. Selected data elements routinely captured by ANZDATA will be utilized for trial purposes, including baseline participant characteristics, acute rejection, graft function, graft survival, and patient survival. Data on donor characteristics will be collected through linkage with the Australia and New Zealand Organ Donation Registry (ANZOD), which is administered and managed by ANZDATA. Data elements collected specifically for the BEST-Fluids trial are directly entered into the registry via an additional trial data collection module. REDCap (Research Electronic Data Capture), a secure, web-based software platform designed to support data capture for research studies [44] is used to collect data on protocol deviations and serious adverse events (SAEs), as well as sub-study data.

### Confidentiality {27}

Any information that may identify a participant will be excluded from the data presented in the public arena. All study-related information is stored securely at each study site, and local databases are secured with password-protected access systems. All copies of clinical records, reports, data collection, process, and administrative forms are identified by a coded identification number only.

Participant information submitted to the ANZDATA Registry for this trial includes individual identifiers and demographic details, and along with routine non-trial registry data collection, is stored on secure servers at the South Australian Health and Medical Research Institute (SAHMRI) in Adelaide, Australia. ANZDATA acts in accordance with Australian and New Zealand national privacy principles and has detailed policies and procedures for data management to safeguard privacy and confidentiality (see https://www.anzdata.org.au/anzdata/services/data-policies/). Staff at study sites have access to identified information about their site’s participants held in ANZDATA for the purpose of data entry and query resolution during the trial. Staff at the AKTN trial coordinating center only have access to de-identified trial data.

### Plans for collection, laboratory evaluation, and storage of biological specimens for genetic or molecular analysis in the trial/future use {33}

No biological specimens are being collected in this study.

## Statistical methods

### Statistical methods for primary and secondary outcomes {20a}

The effect of treatment, Plasmalyte versus 0.9% saline, on the primary outcome of DGF will be analyzed using a log-binomial regression model, with treatment group, three minimization variables (donor status (DBD, DCD), machine perfusion (no, yes), KDRI tertile), and ischemic time [8–10] as predictor variables (fixed effects) in the model, and study center as a random effect. Sensitivity analyses will adjust for potential confounding due to any baseline characteristics that are substantially unbalanced between the treatment groups.

The effect of treatment on secondary outcomes will be analyzed using statistical methods appropriate for the type of outcome. Secondary outcomes are variously binary, ordinal, count, and time to event. Binary secondary outcomes will be analyzed using log-binomial regression. Ordinal outcomes will be analyzed using the Wilcoxon rank-sum test and ordinal logistic regression. Count outcomes will be analyzed using Poisson regression models. If there is under-dispersion or over-dispersion, residuals will be examined to determine the most suitable model (e.g. quasi-Poisson regression, negative binomial regression). Continuous outcomes will be compared using linear regression or an appropriate non-parametric test if assumptions are not met. Mortality and graft survival will be analyzed using Kaplan-Meier curves, the log rank test, and Cox regression.

Analyses will be by intention to treat; participants will be included in the intention-to-treat analysis if they are randomized and receive a deceased donor kidney transplant within 48 h of randomization. Participants whose transplant does not proceed will be excluded from analysis of the primary and secondary outcomes, although those who receive trial fluids will be included in analysis of safety outcomes. Adverse events will be tabulated by treatment group. Depending on the amount and type of data, differences between treatment groups in adverse events may be tested by binary logistic or Poisson regression. All analyses will be described in a detailed statistical analysis plan made publicly available before data analysis commences and treatment allocations are un-blinded.

### Interim analyses {21b}

There are no formal interim analyses planned.

### Methods for additional analyses {20b}

A cost-effectiveness analysis of the interventions will be conducted from a health funder perspective, with the hypothesis that using Plasmalyte rather than 0.9% saline will be a dominant intervention, i.e. will be both more effective and less costly. Information on health-related quality of life derived from the EQ-5D questionnaires will be used to calculate utilities and quality-adjusted life years (QALYs). Resource use for the index transplantation will include the costs of fluids, hospitalizations, dialysis, laboratory tests, hospital clinics, physician visits, procedures, prescribed treatments, and medications. In addition, over the 12 months, data on healthcare resource use will be obtained for Australian participants via linkage to the Admitted Patient Data Collection in each state, and Medicare Australia for outpatient health service use for Australian patients (medical and pharmaceutical - Medicare Benefits Schedule (MBS), and the Pharmaceutical Benefits Scheme (PBS)). For New Zealand participants, data on resource use will be obtained via linkage using the National Health Index number with the national data collections (including the National Minimum Dataset, Mortality Collection, Laboratory Claims Collection, Pharmaceutical Collection, and others as appropriate) held by the Ministry of Health. Unit costs will be obtained from the most recent and relevant Australian-Refined Diagnosis Related Group costs (AR-DRG) and MBS and PBS costs.

Incremental cost-effectiveness ratios (ICERs) will be calculated as costs per life year and QALY gained at 12 months using the mean costs and health outcomes in the Plasmalyte group compared with the 0.9% saline group. Results will be plotted on a cost-effectiveness plane, with bootstrapping used to estimate the distributions of costs and health outcomes, to estimate confidence intervals for the ICERs, and to generate cost-effectiveness acceptability curves. Sensitivity analyses will be explored.

### Methods in analysis to handle protocol non-adherence and any statistical methods to handle missing data {20c}

Analyses will be performed on the intention-to-treat population (as described above). An adherence-adjusted analysis will be performed on the primary outcome if there is more than 4% non-adherence to treatment or differential adherence across treatments. All analyses, including any methods used to handle missing data, will be described fully in the pre-specified statistical analysis plan.

### Plans to give access to the full protocol, participant-level data, and statistical code {31c}

The full trial protocol and the statistical code used for analysis will be made publicly available after publication of the primary results of the trial. Individual participant data that underlie the results reported in the primary publication, after de-identification (text, tables, figures and appendices), will be available for individual participant data meta-analysis, beginning 2 years and ending 5 years after the main publication. Proposals may be submitted up to 5 years after article publication. After 5 years, the data will be available in our University’s data warehouse but without investigator support other than deposited metadata. An independent review board will assess proposals based on the following criteria: sound science, benefit-risk balancing, and research team expertise.

## Oversight and monitoring

### Composition of the coordinating committee and Trial Steering Committee {5d}

The coordinating committee includes the co-chairs of the Trial Steering Committee (TSC) and a member of the AKTN executive committee as a deputy chair. Members include a subset of TSC members, AKTN’s project management staff and the AKTN statistician, with additional expertise co-opted as required (e.g. Registry staff or health economists). The committee is responsible for oversight and direction on operational aspects of the study, and provides regular reports and secretariat support to the TSC.

The TSC is co-chaired by the lead Principal Investigators for the study and includes AKTN executive members, AKTN project management staff, ANZDATA Registry staff, nephrologist investigators from adult and pediatric hospitals, an anesthetist, surgeon, transplant nurse practitioner, statistician, and two health economists. The TSC is responsible for obtaining funding (including grant writing as required), drafting and amending the protocol and other key trial documents, monitoring recruitment, data entry completion and quality, and drafting and approving the statistical analysis plan and other trial publications. The TSC receives reports from the Data Safety Monitoring Board (DSMB) and is responsible for decisions concerning the continuation, modification or termination of the trial following recommendations from the DSMB.

### Composition of the data monitoring committee, its role, and reporting structure {21a}

An independent DSMB has been formed and operates in accordance with a trial-specific DSMB Charter. Members have no financial or scientific conflicts of interest with the BEST-Fluids trial. The DSMB chair is a clinician with extensive clinical trials and DSMB experience. The DSMB statistician is an experienced clinical trials statistician with extensive DSMB experience. Additional members are clinicians with clinical trials and DSMB experience.

The DSMB remit is to protect the safety of trial participants and the scientific integrity of the trial by monitoring the accumulating safety and operational data, and meets approximately every 6 months during the trial. There are no formal statistical guidelines for early stopping. Following each of its meetings to date (as of March 2020), the DSMB has recommended that the trial continue without changes to the trial protocol. Under the DSMB charter, the investigators retain sole decision-making responsibility for modifications to, or early stopping of, the trial.

### Adverse event reporting and harms {22}

Serious adverse events (SAEs) that are considered related to study treatment are monitored and reported up until day 7 post-transplant. Study-specific reportable adverse events, including hyperkalemia (up to day 7), dialysis (up to week 12), death, acute rejection episodes, and graft loss, are also being monitored during the trial.

In addition to including details of SAEs and study-specific reportable adverse events reported by study sites, reporting for the DSMB was modified in 2019 after a request for additional safety data, to include administrative coding data from hospital discharge summaries to allow tabulation of discharge diagnoses, complications, and procedure codes recorded during the index transplant admission for each participant. These data are extracted periodically from clinical records by local hospital coding staff at each site and submitted to the AKTN for inclusion in DSMB safety reports. In the context of this pragmatic trial, this measure was added to enable capture of any unanticipated adverse events and thereby ensure sufficient monitoring of trial participant safety.

### Frequency and plans for auditing trial conduct {23}

GCP compliance monitoring is being conducted by the Sponsor. Risk-based monitoring is in place for the study.

### Plans for communicating important protocol amendments to relevant parties {25}

All protocol amendments will be approved by responsible independent ethics committees and local site governance prior to implementation. Trial registration information has been updated with the amendment information. No amendments have been made to the protocol that have required re-consent from participants.

## Dissemination plan {31a}

Trial findings will be disseminated at national and international scientific meetings, by publication in a scientific journal, and through submission of the results to trial registration databases. Authorship for all trial publications will be based on criteria formulated by the International Committee of Medical Journal Editors, available at www.icmje.org.

Additionally, results will be disseminated to trial participants, study staff, clinicians, and patient groups via direct approaches, and a variety of traditional and electronic media, including newsletters, on social media and the AKTN website.

## Discussion

Interventions that reduce the impact of delayed graft function (DGF) in kidney transplantation have the potential to improve transplant outcomes and reduce costs. This multi-center, prospective, randomized, double-blind, pragmatic trial has been designed to determine whether the type of fluid therapy used peri-operatively in transplantation is important, specifically whether 0.9% saline given to a recipient exacerbates ischemia-reperfusion-injury-associated acute kidney injury and DGF, and whether using a balanced low-chloride alternative could prevent these adverse effects. Given that IV fluid therapy is both inexpensive and a critically important aspect of kidney transplant care, evidence for the superiority of Plasmalyte over 0.9% saline from this trial would provide strong justification for a change in clinical practice to using balanced crystalloids as the standard of care. Given the relatively large sample size and the expected generalizability of results to other transplant populations, a finding of no significant difference between the groups for the primary and key secondary outcomes with clinically important differences outside the bounds of the confidence intervals for effect estimates would imply that any benefit with balanced low-chloride crystalloids over 0.9% saline either does not exist, or is not clinically important for the majority of deceased donor kidney transplant recipients. Similarly, although the results of previous trials would make such findings unexpected, better outcomes with 0.9% saline would justify continuing with this type of fluid as the standard of care. Regardless, BEST-Fluids will provide important data on the relative safety of these two fluid types, particularly in relation to any electrolyte abnormalities, such as hyperkalemia, or other peri-operative complications.

In keeping with the pragmatic study design, and to maximize the generalizability of the study findings, eligibility criteria were designed to enroll a broad range of deceased donor kidney transplant recipients, with an estimated incidence of DGF of 30–40%. It is estimated that > 95% of deceased donor transplant recipients are eligible for this trial. Trial processes have been designed to be integrated into routine clinical care at participating hospitals, with multiple pragmatic aspects enabling transplant clinicians to easily enroll their patients without necessarily requiring the presence of research staff, and to prescribe the trial fluid intervention with maximum flexibility while maintaining a blinded treatment allocation. All data collected, apart from the quality of life questionnaires, are either already routinely collected or readily available in the clinical record; no additional laboratory tests or procedures are required for the trial.

By using a registry-based trial design, we sought to align trial processes with clinical practice using established registry data collection practices. Registry trial designs have a number of potential advantages in terms of increased efficiency, lower costs, alignment with standard clinical care, and reduction in investigator burden [45]. Staff at participating hospitals in BEST-Fluids are familiar with ANZDATA and the requirements of its data collection; 100% of renal units in Australia and New Zealand contribute data on patients treated with a kidney transplant. A purpose-built point-of-care data entry system for ANZDATA was rolled out to staff in renal units at around the time that recruitment commenced in the BEST-Fluids study; trial processes including enrolment, randomization and data collection have been incorporated into this system with the use of an additional integrated software module.

There are two other significant advantages of using the registry-based design. First, because ANZDATA contains data on all kidney transplant recipients in Australia and New Zealand, it will be possible to directly compare BEST-Fluids participants with the population of transplant recipients who are non-participants (who either had their transplant at a center not involved in the trial or who underwent transplantation at a participating hospital but are not enrolled for other reasons). This will enable an assessment of external validity and generalizability of the study findings through a comparison of patient characteristics and outcomes between these two groups. Second, all participants will continue to have data submitted to ANZDATA by local renal unit staff as per usual practice for death, graft failure, and other outcomes after the 12-month trial follow up ends, which will facilitate long-term follow up of trial participants at minimal cost.

An important consideration in the design for this trial was whether or not to exclude patients found to have hyperkalemia prior to enrolment. Pre-operative hyperkalemia is a not infrequent occurrence in patients with ESKD who are admitted for surgery, including transplantation. Some previous trials of low-chloride solutions versus 0.9% saline in transplant recipients have excluded patients with hyperkalemia (pre-operative serum potassium > 5.5 mmol/L) due to concerns about the potassium content of low-chloride fluids (Plasmalyte, Ringers Lactate, and/or Elomel Isoton) [24]. Additionally, the manufacturer of Plasmalyte recommends caution with its use in patients with hyperkalemia [46]. However, these concerns have not been borne out in studies to date, which have not demonstrated increased hyperkalemia in patients receiving balanced low-chloride solutions. In the Weinberg trial [29], there was significantly less hyperkalemia in the Plasmalyte group, despite this study only excluding patients with untreated pre-operative potassium > 6.0 mmol/L. It therefore seems likely that Plasmalyte does not cause clinically significant hyperkalemia despite its potassium content (5 mmol/L, similar to plasma). Moreover, the low-chloride concentration may confer a protective effect, even in the setting of poor graft function and oliguria, due to avoidance of hyperchloremic acidosis combined with the inclusion of gluconate and acetate anions as bicarbonate precursors. Potential trial participants with pre-operative hyperkalemia are therefore not excluded from BEST-Fluids, which is consistent with the pragmatic approach to eligibility criteria and will ensure maximum applicability of the trial findings. The protocol recommends that treating physicians actively manage and treat pre-operative hyperkalemia > 6.0 mmol/L prior to transplant surgery (as would be considered usual clinical practice), thereby minimizing the potential risk. Hyperkalemia will be reported as an important secondary outcome for the trial.

In the protocol for BEST-Fluids originally approved by ethics committees and in operation at the time enrolment commenced in January 2018 (protocol version 2.0, 13 June 2017), the primary outcome measure was Early Kidney Transplant Function*;* a novel outcome measure incorporating two commonly used measures of DGF: (1) the duration of DGF in days in participants requiring dialysis and (2) the rate of transplant graft function recovery measured by creatinine reduction ratio on day 2 (CRR2) [38] for participants who do not receive dialysis. After a review of the rates of recruitment, protocol adherence and DGF in the first 113 trial participants, the study protocol was amended in 2018 (Protocol version 3.0, 22 October 2018) to change the primary outcome to a binary outcome measure based simply on the incidence of DGF (defined as dialysis within 7 days of transplant) and increase the sample size from the original number of 574 to the current sample size of 800 participants. These changes were made to align the trial primary outcome with other, similar contemporary trials of interventions for DGF, and to make it easier for clinicians to interpret the trial results, with the ultimate goal of facilitating a rapid translation of the trial findings into practice. The original primary outcome has been retained as a key secondary outcome measure; further details of this outcome measure and the original sample size calculation are provided in Additional file 4.

A particular strength of BEST-Fluids is its randomized, blinded design, with the use of minimization to ensure balance between treatment groups for relevant factors that impact the risk of the primary outcome of DGF. Blinding is particularly important for avoidance of bias in a trial of this type, where the primary outcome (DGF, the requirement for dialysis in the first post-transplant week) involves a clinical decision made by physicians caring for the patient. Furthermore, there is significant heterogeneity in the incidence of DGF between centers, even after adjustment for patient-level and center-level characteristics, suggesting that local transplant practices and clinician propensity to utilize dialysis during the post-transplant period vary considerably [41].

There are some limitations that need to be considered. By design, BEST-Fluids does not mandate any specific approaches to the volume of IV fluid therapy to use either during surgery or in the post-operative period, and fluid volume may be an important risk factor for the development of DGF. In a recent trial of patients undergoing major abdominal surgery, a liberal approach to fluid therapy was associated with a reduced risk of acute kidney injury compared with a more restrictive approach, in contrast to previous studies that had suggested that liberal fluid therapy was associated with inferior outcomes due to tissue edema, fluid overload, and other post-operative complications [47]. Data on fluid volume and weight change are being collected in BEST-Fluids. Both the duration of cold ischemic time (from retrieval until implantation commences) and warm ischemic time (from rewarming to reperfusion of the graft following completion of vascular anastomoses) are major factors that affect the risk of DGF [9, 10], which are not incorporated into the randomization allocation algorithm as this information is not available at the time of enrolment into the study. However, the primary analysis of the effects of treatment on the primary outcome will be adjusted for total ischemic time, in addition to the minimization variables. Additional pre-specified analyses of the primary and key secondary outcomes will include separate adjustment for cold and warm ischemic times, fluid volume, and change in weight as a measure of fluid overload.

In conclusion, BEST-Fluids is an ongoing randomized, controlled, pragmatic, registry-based trial that will provide the most definitive comparative effectiveness data to date on DGF and other important clinical outcomes, using Plasmalyte versus 0.9% saline in deceased donor kidney transplantation. If the hypothesis that Plasmalye is superior to 0.9% saline is proven, this will provide an important and inexpensive means to improve transplant outcomes, which can be rapidly implemented into clinical practice.

## Trial status

**Table:** 

Protocol version	3.1
Protocol date	2 March 2020
Recruitment start date	30 January 2018
Anticipated recruitment end date	31 December 2020

## Supplementary information

**Additional file 1.** Pragmatism assessment of the trial design. PRECIS-2 scores for trial domains in the BEST-Fluids trial.

**Additional file 2.** List of active study sites. Includes list of all sites in the study which are recruiting participants.

**Additional file 3.** Protocol recommendations for concurrent participant management.

**Additional file 4.** Early kidney transplant function - secondary outcome measure (1) and original primary outcome measure. Includes description of this composite ranked outcome measure and details of the original sample size calculation.

**Additional file 5.** Schedule of Assessments. Outlines the study visits and the assessments and procedures at each study visit, including details of the timing of visits between baseline and day 2.

**Additional file 6.** Participant information sheet and consent form.

## Data Availability

The datasets generated and/or analyzed during the current study (individual participant data that underlie the results reported in the primary publication, after de-identification) will be available from the corresponding author on reasonable request for individual participant data meta-analysis, beginning 2 years following the main publication.

## Abbreviations

AKTN: Australasian Kidney Trials Network
ANZDATA: Australia and New Zealand Dialysis and Transplant Registry
AR-DRG: Australian Refined Diagnosis Related Groups
CKD-EPI: Chronic Kidney Disease - Epidemiology Collaboration
CI: Confidence interval
CRR2: Creatinine reduction ratio on day 2
DSMB: Data and Safety Monitoring Board
DCD: Donation after circulatory death
DBD: Donation after brain death
DGF: Delayed graft function
eGFR: Estimated glomerular filtration rate
EQ-5D-5L: EuroQol five dimensions, 5 levels questionnaire
EQ-5D-Y: EuroQol five dimensions, Youth questionnaire
ESKD: End-stage kidney disease
GCP: Good Clinical Practice
ICERs: Incremental cost-effectiveness ratios
IV: Intravenous
KDRI: Kidney Donor Risk Index
MBS: Medicare Benefits Schedule
PBS: Pharmaceutical Benefits Schedule
PRECIS-2: Pragmatic-Explanatory Continuum Indicator Summary 2
REDCap: Research Electronic Data Capture
RR: Relative risk
QALY: Quality-adjusted life years
SAE: Serious adverse event
TSC: Trial Steering Committee
