# Supplementary material for: Study Protocol for Better Evidence for Selecting Transplant Fluids (BEST-Fluids): a pragmatic, registry-based, multi-center, double-blind, randomized controlled trial evaluating the effect of intravenous fluid therapy with Plasma-Lyte 148 versus 0.9% saline on delayed graft function in deceased donor kidney transplantation
Source: Trials. 2020 May 25;21:428. doi: 10.1186/s13063-020-04359-2 (PMC7249430; doi:10.1186/s13063-020-04359-2)
Supplement: Supplementary file 1 — Additional file 1. Pragmatism assessment of the trial design. PRECIS-2 scores for trial domains in the BEST-Fluids trial. [file 13063_2020_4359_MOESM1_ESM.docx]

Additional File 1: Pragmatism assessment of the trial design. PRECIS-2 scores for trial domains in the BEST-Fluids trial [1]

Pragmatic trials**^[[1]](#footnote-1)^** set out to answer the question *“Does this intervention work under usual conditions?”* Such trials contrast with traditional, more explanatory trials that seek to answer the question: *“Can this intervention work under ideal conditions?”* PRECIS-2 is a validated tool for assessing how pragmatic a trial is. It uses nine domains - eligibility criteria, recruitment, setting, organization, flexibility (delivery), flexibility (adherence), follow-up, primary outcome, and primary analysis - scored using a 5-point Likert scale as follows:

1. Very explanatory
2. Rather explanatory
3. Equally pragmatic and explanatory
4. Rather pragmatic
5. Very pragmatic

See [www.precis-2.org](http://www.precis-2.org) for further details. Scores for each of these domains for BEST-Fluids, and the rationale for each assigned score, are outlined in the table below.

| Item | Domain | Score | Rationale |
| --- | --- | --- | --- |
| 1 | **Eligibility criteria** Who is selected to participate in the trial? | 4 | All patients admitted for a deceased donor kidney transplant are eligible for BEST-Fluids, apart from multi-organ recipients, children <20kg and those with a known hypersensitivity to one of the interventions. The likely eligible population includes >95% of people receiving with a deceased donor kidney transplant. |
| 2 | **Recruitment**  How are participants recruited into the trial? | 5 | Participants are recruited into the BEST-Fluids trial in the usual care setting, i.e. at the time of admission for a deceased donor kidney transplant. |
| 3 | **Setting** Where is the trial being done? | 5 | BEST-Fluids is being conducted at multiple hospitals which perform deceased donor kidney transplantation in Australia and New Zealand, including centers with large and small transplant programs, academic and non-academic centers, and adult and pediatric hospitals. |
| 4 | **Organization** What expertise and resources are needed to deliver the intervention? | 4 | Staff delivering the intervention (trial fluid therapy) are the usual staff providing clinical care to participants (i.e. physicians and resident medical staff, anesthetists, recovery and ward nursing staff). They are provided with information about the trial and an initial training session, and periodic refresher updates from research staff. During delivery of the intervention, assistance is available to clinical staff from trial research staff. |
| 5 | **Flexibility (delivery)** How should the intervention be delivered? | 3 | Apart from specifying that the intervention (trial fluids) be used for all standard fluid therapy purposes (in place of open label fluids) until 48 hours post-transplant, fluid therapy is prescribed and administered as per usual care, with clinical staff having discretion on the rate and volume of fluid given, and when it should be discontinued. The protocol includes specific directions on some concurrent interventions directly related to the intervention (e.g. restricting open label fluid therapy) but does not prevent clinicians from utilizing these if required. There are no additional interventions that would not occur outside usual care. Complications and side effects are managed as per standard care. Trial specific fluid administration sheets, labels on medication charts, and information sheets on the trial in the clinical records are used to improve adherence. |
| 6 | **Flexibility (adherence)** What measures are in place to ensure participants adhere to the intervention? | 5 | No measures are in place to improve participant adherence to the intervention. |
| 7 | **Follow-up** How closely are participants followed up? | 3 | Participants have no additional visits in addition to usual care. The only additional data collected for the trial from participants are measures of health-related quality of life (EQ-5D questionnaires); participants are contacted at visit time points to complete these, and reminders may be sent if required. All clinical, laboratory and health resource use data required for the trial are collected from clinical records and administrative databases. The ANZDATA registry is used for data collection, similar to usual care. |
| 8 | **Primary outcome** How relevant is it to participants? | 4 | Dialysis within 7 days of transplant is the standard measure of delayed graft function (DGF) and considered a significant adverse complication of deceased donor kidney transplantation. For participants, needing to have dialysis means having one or more additional invasive treatments with a risk of complications and side effects, so this outcome has direct relevance to them following transplant surgery. Determining DGF requires no central adjudication or additional testing. However, DGF is a short-term outcome reflecting early complications and of less importance than other long-term outcomes, such as patient or graft survival. |
| 9 | **Primary analysis** To what extent are all data included? | 5 | An intention to treat analysis including all available data will be used for analysis of the primary outcome. |

**Reference**

1. Loudon K, Treweek S, Sullivan F, Donnan P, Thorpe KE, Zwarenstein M: The PRECIS-2 tool: designing trials that are fit for purpose. *BMJ* 2015, 350:h2147.

1. The text describing PRECIS-2 here is adapted from the paper describing PRECIS-2 by Loudon et al (2015). [↑](#footnote-ref-1)
