# Supplementary material for: Study Protocol for Better Evidence for Selecting Transplant Fluids (BEST-Fluids): a pragmatic, registry-based, multi-center, double-blind, randomized controlled trial evaluating the effect of intravenous fluid therapy with Plasma-Lyte 148 versus 0.9% saline on delayed graft function in deceased donor kidney transplantation
Source: Trials. 2020 May 25;21:428. doi: 10.1186/s13063-020-04359-2 (PMC7249430; doi:10.1186/s13063-020-04359-2)
Supplement: Supplementary file 2 — Additional file 2. List of active study sites. Includes list of all sites in the study which are recruiting participants. [file 13063_2020_4359_MOESM2_ESM.docx]

**Additional File 2: List of active study sites** (in order of activation)

| **New Zealand** |
| --- |
| Auckland City Hospital, Auckland |
| Christchurch Hospital, Christchurch |
| Wellington Hospital, Wellington |
| Starship Children's Hospital, Auckland |
| **Australia** |
| Royal Prince Alfred Hospital, NSW |
| Princess Alexandra Hospital, QLD |
| Austin Health, VIC |
| Royal Adelaide Hospital, SA |
| Westmead Hospital, NSW |
| Prince of Wales Hospital, NSW |
| Fiona Stanley Hospital, WA |
| Monash Medical Centre, VIC |
| St Vincent's Hospital Melbourne, VIC |
| Queensland Children's Hospital, QLD |
| Sydney Children's Hospital, NSW |
| Sir Charles Gairdner Hospital, WA |
