# Supplementary material for: Study Protocol for Better Evidence for Selecting Transplant Fluids (BEST-Fluids): a pragmatic, registry-based, multi-center, double-blind, randomized controlled trial evaluating the effect of intravenous fluid therapy with Plasma-Lyte 148 versus 0.9% saline on delayed graft function in deceased donor kidney transplantation
Source: Trials. 2020 May 25;21:428. doi: 10.1186/s13063-020-04359-2 (PMC7249430; doi:10.1186/s13063-020-04359-2)
Supplement: Supplementary file 3 — Additional file 3. Protocol recommendations for concurrent participant management. [file 13063_2020_4359_MOESM3_ESM.docx]

**Additional File 3: Protocol recommendations for concurrent participant management**

### Non-trial (open-label) fluid therapy

The use of other non-trial fluids such as blood products will be permitted as per routine clinical care and treating physician discretion. However, the use of non-trial crystalloids is discouraged except under specific circumstances detailed below. If non-trial crystalloids are used, blinded trial solutions should be continued concurrently wherever possible.

In all situations where open-label non-trial fluids are used, the specific indications for these and the volume administered will be recorded.

Permitted indications for non-trial crystalloids include:

- Hypotonic solutions (5% dextrose, 4% dextrose/0.18% saline, 3% dextrose/0.3% saline, 0.45% saline, or sterile water)
  - Significant polyuria (>500mL/hour urine output for >4 hours), which in the opinion of the treating physician:
    - may be related to the composition of the blinded isotonic fluid (Plasmalyte or 0.9% saline), AND
    - may lead to sodium and/or volume overload, AND
    - cannot be modified by altering the rate of blinded fluid administration.
  - Hypernatremia (serum sodium ≥146 mmol/L)

Hypotonic solutions used for these indications should be administered no more frequently than a ratio of 1:2 open-label hypotonic solution to blinded trial crystalloids.

- Diabetes management

When used as part of local standard of care, open-label non-trial crystalloid solutions can be administered together with an insulin infusion, however, blinded fluids should be used for maintenance fluid purposes wherever possible. *It is recommended that insulin be reconstituted in a low volume of 0.9% saline or 5% dextrose*, e.g. 100 units of insulin in 100-500 mL of 0.9% saline and administered at an appropriate infusion rate per hour based on glucose monitoring. A bolus of dextrose (e.g. 10% or 50% dextrose) can be administered at any time for the management of hypoglycemia.

- Intravenous (IV) drug administration

IV drug therapies should be administered in appropriate solutions as per manufacturer recommendations or approved local practice. Blinded fluids should continue.

Colloids and blood products

Use of IV albumin and other colloids is permitted but strongly discouraged unless there is compelling clinical indication. Blinded fluids should be continued. Blood products can be administered at any time as clinically indicated.

Targeted strategies

On the basis that there is currently little evidence of efficacy or benefit for targeted strategies for selecting and manipulating fluid therapy (e.g. adjusting IV fluids used on the basis of serum pH, urine sodium, etc.), it is not recommended that such approaches be used.

### Hyperkalemia

Hyperkalemia should be treated as per standard practice i.e. with glucose/insulin, beta agonists, ion-exchange resins, calcium gluconate and/or dialysis where required. Pre-operative hyperkalemia where the serum potassium is >6.0 mmol/L should be actively treated prior to transplant surgery as appropriate; this may include pre-operative dialysis.

Blinded fluids should be continued regardless of hyperkalemia and administered routinely for maintenance, replacement or resuscitation purposes. Where clinically appropriate (e.g. oliguria/anuria with adequate volume status/fluid overload), blinded fluids should be temporarily or permanently withheld to avoid the risk of developing adverse effects from volume overload, rather than hyperkalemia per se.

### Dialysis

Dialysis should be performed when clinically indicated as per clinicians caring for the patient; the indications for dialysis (e.g. uremia, fluid/volume overload, hyperkalemia) will be recorded. Dialysis treatments (hemodialysis, hemodiafiltration, sustained low efficiency daily dialysis, any form of continuous renal replacement therapy, or peritoneal dialysis) will be prescribed by treating physicians as per standard local practice. Where intravenous fluid boluses are required during a dialysis treatment within the intervention fluid period (up to 48 hours post-transplant), open label crystalloid fluids may be administered as per standard care in line with local practice and protocols.

### Completion of trial fluid intervention

Once the 48-hour post-transplant time-point is reached on day 2, participants requiring continued intravenous fluids may be converted to open label fluids as prescribed by the treating physician. Blinded fluids may be continued beyond 48 hours until the allocated stock is exhausted per investigator/treating physician discretion.

### Other transplant management

All participants will receive usual transplant management as per local standard of care. This includes peri-operative anesthesia and invasive monitoring, circulatory support, surgical care, immunosuppression, routine prophylactic measures, and management of fluid overload, hyperkalemia and other electrolyte disturbances, and other complications.
