# Supplementary material for: Study Protocol for Better Evidence for Selecting Transplant Fluids (BEST-Fluids): a pragmatic, registry-based, multi-center, double-blind, randomized controlled trial evaluating the effect of intravenous fluid therapy with Plasma-Lyte 148 versus 0.9% saline on delayed graft function in deceased donor kidney transplantation
Source: Trials. 2020 May 25;21:428. doi: 10.1186/s13063-020-04359-2 (PMC7249430; doi:10.1186/s13063-020-04359-2)
Supplement: Supplementary file 4 — Additional file 4. Early kidney transplant function - secondary outcome measure (1) and original primary outcome measure. Includes description of this composite ranked outcome measure and details of the original sample size calculation. [file 13063_2020_4359_MOESM4_ESM.docx]

**Additional File 4: Early kidney transplant function – secondary outcome measure (1) and original primary outcome measure** (prior to Protocol versions 3.0 and later)

In the original protocol for the BEST-Fluids trial that was in operation at the time enrolment commenced in January 2018 (protocol version 2.0, 13 June 2017), the primary outcome measure was *Early Kidney Transplant Function*; a ranked composite of duration of delayed graft function (DGF) and the rate of kidney transplant function recovery. In the current protocol (version 3.0, October 2018), this measure is now a key secondary outcome measure.

Early Kidney Transplant Function (EKTF) was proposed as a novel outcome measure incorporating two commonly used measures of delayed graft function: (1) the duration of delayed graft function in days for participants requiring dialysis, and (2) the rate of transplant graft function recovery measured by creatinine reduction ratio on day two (CRR2) [1] for participants who do not receive dialysis.

EKTF is determined as follows (see Figure A1):

1. For participants who do not need dialysis, kidney transplant function measured with *creatinine reduction ratio (CRR2) on post-transplant day 2* [CRR2 (%) = ([creatinine_day1_-creatinine_day2_]*100) / creatinine_day1_] [1] is ranked from best to worst (smaller = worse)
2. For participants who require dialysis within 7 days post-transplant, the *time from transplant to the final dialysis treatment* (days) will be ranked from best to worst (longer = worse)
3. The two sets of ordered data are combined into a single ranking of best to worst graft function, where the largest increase in CRR2 is ranked highest (best outcome), and the longest dialysis duration (or failure to recover kidney transplant function by 12 weeks) is ranked lowest (worst outcome)

EKTF was proposed as a novel composite outcome measure to use in this trial because it incorporates an important post-transplant health outcome of relevance to clinicians and patients (dialysis requirement), and validated surrogate measures of long-term graft outcome (both measures). This approach was intended to ensure relevance to the research question *and* to clinical practice, incorporate accepted measures of therapy effectiveness, be able to be observed in all patients, be objective, and improve trial efficiency. Specifically:

1. Dialysis post-transplant leads to significant patient morbidity, reduced quality of life and increased costs [2], and is a strong predictor of graft loss and death [3];
2. CRR2 is more sensitive (but less specific) for poor graft outcomes than requirement for dialysis [4, 5]. CRR2 <30% is a predictor of intermediate risk for graft failure and reduced graft function compared to dialysis-DGF [1, 6];
3. Both components of the composite are valid when used continuously as well as categorically. Prolonged dialysis duration post-transplant is associated with inferior graft survival [7, 8] and CRR2 predicts subsequent long-term graft function [1, 6];
4. Efficiency and feasible sample size. A continuous ranked outcome enables the trial to be conducted with a more efficient sample size. A similar ranked composite primary outcome construct was used in a hemodialysis trial (Chertow et al; *New Engl J Med* 2010) with substantial benefits for trial efficiency [9].

After a review of the rates of recruitment, protocol adherence and the incidence of DGF in the first 113 trial participants, the study protocol was amended in 2018 (Protocol version 3.0) to increase the sample size from the original number of 574 to a revised sample size of 800 participants, and to change the primary outcome to a binary outcome measure based simply on the incidence of DGF (defined as dialysis within 7 days of transplant). These changes were made to align the trial primary outcome with other, similar contemporary trials of interventions for DGF, and make it easier for clinicians to interpret the trial results, with the ultimate goal of ensuring rapid translation of trial findings into practice. EKTF has been retained as the first secondary outcome measure.

**Original sample size calculation**

The original sample size (574 participants; Protocol version 2.0) was based on a comparison of two independent groups using Noether’s method for the ordinal (ranked) primary outcome [10] of Early Kidney Transplant Function, and analyzed with a Wilcoxon rank sum test. It was estimated that a clinically meaningful difference in the primary outcome would be that at least 15% of participants in the Plasmalyte group are found to be better off, i.e. ranked higher than participants in the saline group, either by requiring less dialysis (or no dialysis at all), or by having quicker recovery of transplant graft function; both measures reflecting improved graft function overall. A sample size of 574 participants (287 per group), has 80% power to show this difference, with a two-sided alpha of 5%, allowing for 2.5 % non-adherence and up to 10% loss to follow-up.


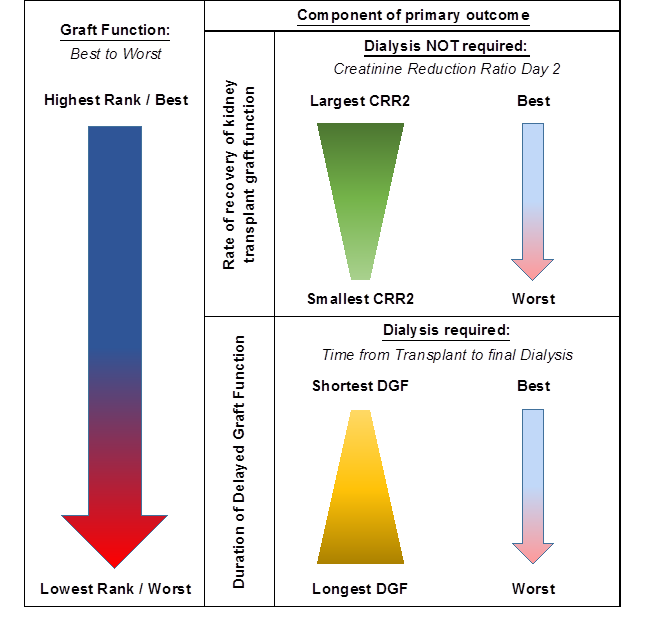


***Figure A1. Early Kidney Transplant Function: outline of the components of the ranked composite outcome measure (secondary outcome 1).***

**References**

1. Rodrigo E, Ruiz JC, Pinera C, Fernandez-Fresnedo G, Escallada R, Palomar R, Cotorruelo JG, Zubimendi JA, Martin de Francisco AL, Arias M: Creatinine reduction ratio on post-transplant day two as criterion in defining delayed graft function. *Am J Transplant* 2004, 4(7):1163-1169.

2. Hagenmeyer EG, Haussler B, Hempel E, Grannas G, Kalo Z, Kilburg A, Nashan B: Resource use and treatment costs after kidney transplantation: impact of demographic factors, comorbidities, and complications. *Transplantation* 2004, 77(10):1545-1550.

3. Butala NM, Reese PP, Doshi MD, Parikh CR: Is delayed graft function causally associated with long-term outcomes after kidney transplantation? Instrumental variable analysis. *Transplantation* 2013, 95(8):1008-1014.

4. Mallon DH, Summers DM, Bradley JA, Pettigrew GJ: Defining delayed graft function after renal transplantation: simplest is best. *Transplantation* 2013, 96(10):885-889.

5. Hall IE, Reese PP, Doshi MD, Weng FL, Schroppel B, Asch WS, Ficek J, Thiessen-Philbrook H, Parikh CR: Delayed graft function phenotypes and 12-month kidney transplant outcomes. *Transplantation* 2017, 101(8):1913-1923.

6. Vilar E, Varagunam M, Yaqoob MM, Raftery M, Thuraisingham R: Creatinine reduction ratio: a useful marker to identify medium and high-risk renal transplants. *Transplantation* 2010, 89(1):97-103.

7. de Sandes-Freitas TV, Felipe CR, Aguiar WF, Cristelli MP, Tedesco-Silva H, Medina-Pestana JO: Prolonged delayed graft function is associated with inferior patient and kidney allograft survivals. *PLoS One* 2015, 10(12):e0144188.

8. Giral-Classe M, Hourmant M, Cantarovich D, Dantal J, Blancho G, Daguin P, Ancelet D, Soulillou JP: Delayed graft function of more than six days strongly decreases long-term survival of transplanted kidneys. *Kidney Int* 1998, 54(3):972-978.

9. Chertow GM, Levin NW, Beck GJ, Depner TA, Eggers PW, Gassman JJ, Gorodetskaya I, Greene T, James S, Larive B *et al*: In-center hemodialysis six times per week versus three times per week. *N Engl J Med* 2010, 363(24):2287-2300.

10. Noether GE: Sample Size Determination for Some Common Nonparametric Tests. *J Am Stat Assoc* 1987, 82(398):645-647.
