# Supplementary material for: Study Protocol for Better Evidence for Selecting Transplant Fluids (BEST-Fluids): a pragmatic, registry-based, multi-center, double-blind, randomized controlled trial evaluating the effect of intravenous fluid therapy with Plasma-Lyte 148 versus 0.9% saline on delayed graft function in deceased donor kidney transplantation
Source: Trials. 2020 May 25;21:428. doi: 10.1186/s13063-020-04359-2 (PMC7249430; doi:10.1186/s13063-020-04359-2)
Supplement: Supplementary file 5 — Additional file 5. Schedule of Assessments. Outlines the study visits and the assessments and procedures at each study visit, including details of the timing of visits between baseline and day 2. [file 13063_2020_4359_MOESM5_ESM.docx]

**Additional File 5: Schedule of Assessments**

| **Study Phase** | **Transplant and Early Post-operative Phase** | | | | | **Follow-up Phase** | | | |
| --- | --- | --- | --- | --- | --- | --- | --- | --- | --- |
| **Study Visit** | **Baseline** | **Post-Op** | **Day 1** | **Day 2** | **Day 7** | **Day 28** | **Week 12** | **Week 26** | **Week 52** |
| **Visit Number** | **1** | **2** | **3** | **4** | **5** | **6** | **7** | **8** | **9** |
| **Timing of visit** | **On Admission** | **Arrival at recovery room** | **5-9am day after Post-op** | **24 hours after**  **Day 1** | **7 days after Post-Op** | **28 days after Post-Op** | **84 days after Post-Op** | **182 days after Post-Op** | **364 days after Post-Op** |
|  |  |  |  | (± 120 min) | (± 1 day) | (± 7 days) | (± 7 days) | (± 14 days) | (± 14 days) |
| Screening | X |  |  |  |  |  |  |  |  |
| Consent | X |  |  |  |  |  |  |  |  |
| Randomization | X |  |  |  |  |  |  |  |  |
| Participant / donor details | X |  |  |  |  |  |  |  |  |
| Use of Immunosuppressant drugs | X |  |  |  | X |  |  |  |  |
| Requirement for inotropic support |  |  |  | X |  |  |  |  |  |
| Urine output (last 24 hours) |  |  | X | X | X** |  |  |  |  |
| **Study Intervention** |  |  |  |  |  |  |  |  |  |
| Intervention fluid volume |  | X | X | X | X** |  |  |  |  |
| Non-trial fluids type and volume |  | X | X | X | X** |  |  |  |  |
| **Outcomes** |  |  |  |  |  |  |  |  |  |
| Dialysis requirement |  |  |  |  | X | X | X |  |  |
| Serum Creatinine |  | X | X | X | X | X | X | X | X |
| Treatment for hyperkalemia | X | X | X | X | X** |  |  |  |  |
| Biopsy requirement |  |  |  |  | X | X |  |  |  |
| Acute rejection episodes |  |  |  |  | X | X | X | X | X |
| Quality of life: EQ-5D-5L & EQ-5D-Y | X |  |  |  | X | X | X | X | X |
| Mortality and graft survival |  | X | X | X | X | X | X | X | X |
| Serious Adverse Events | X | X | X | X | X |  |  |  |  |
| **Lab assessments** |  |  |  |  |  |  |  |  |  |
| Haemoglobin, Urea, Electrolytes, Blood pH, Potassium |  | X | X | X |  |  |  |  |  |

** Only recorded until 48 hours post-op

### Timing of visits between Baseline and Day 2

**Baseline** (visit 1) will occur at the time of randomization. **Post-op** (visit 2) will occur at the time of arrival to the recovery room after transplant surgery.

For the purposes of subsequent follow-up study assessments, the day that transplant surgery takes place will be designated as day zero. **Day 1** post-transplant will commence at 12.00 am (00:00:00 hours; midnight) on the next consecutive day after arrival to the recovery room (i.e. Post-op, visit 2). Assessments for Day 1 (visit 3 in the Table) will be conducted between 5 and 9 am on Day 1, as per local practice for routine collection of laboratory samples. **Day 2** post-transplant (visit 4) will commence at 12.00 am the day after Day 1. Assessments for Day 2 (visit 4) will be conducted 24 hours +/- 120 minutes after the assessments on Day 1. Study visits from Day 7 (visit 5) onwards will occur as outlined in Table 5.1.

Two examples of the timing of the study visits on Day 1 and 2 in relation to the timing of transplant surgery and the duration of the intervention are shown in Figure A2.


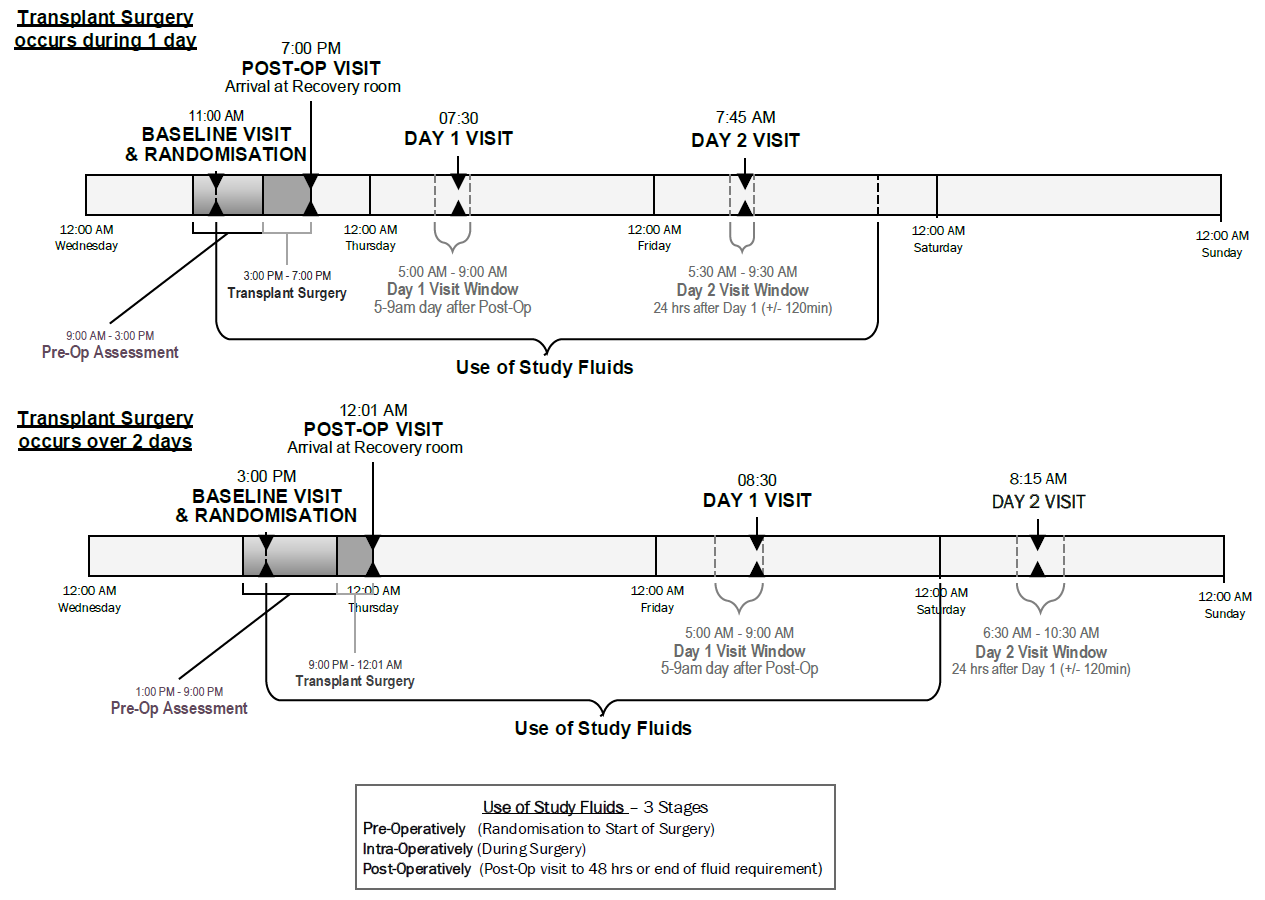


***Figure A2. Examples of the timing of Day 1 and Day 2 visits in relation to transplant surgery and the blinded fluid intervention.***
