# Supplementary material for: Study Protocol for Better Evidence for Selecting Transplant Fluids (BEST-Fluids): a pragmatic, registry-based, multi-center, double-blind, randomized controlled trial evaluating the effect of intravenous fluid therapy with Plasma-Lyte 148 versus 0.9% saline on delayed graft function in deceased donor kidney transplantation
Source: Trials. 2020 May 25;21:428. doi: 10.1186/s13063-020-04359-2 (PMC7249430; doi:10.1186/s13063-020-04359-2)
Supplement: Supplementary file 6 — Additional file 6. Participant information sheet and consent form. [file 13063_2020_4359_MOESM6_ESM.docx]

Additional File 6: Participant Information Sheet and Consent Form

See the following pages for a model Participant Information Sheet and Consent Form used in the BEST-Fluids trial. This is a model of the version used for adults at New Zealand sites.

Participant Information Sheet and Consent forms used in Australia for adults, and the assent and consent forms used for children and parents/guardians respectively in both countries are available from the corresponding author on request.

| **Participant Information Sheet** | Your Letterhead |
| --- | --- |


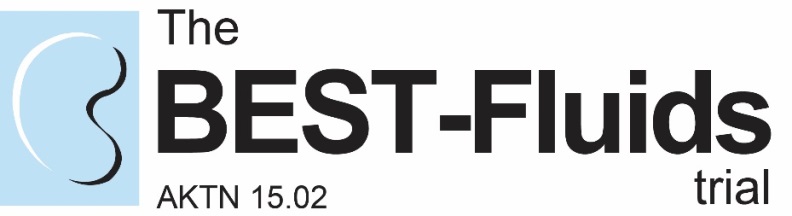


***B***etter ***E***vidence for ***S***electing ***T***ransplant ***Fluids***

An investigator-initiated, pragmatic, registry-based, multi-centre, double-blind, randomised controlled trial evaluating the effect of Plasmalyte versus 0.9% saline on early kidney transplant function in deceased donor kidney transplantation.

*Lead Principal Investigators:*

*Dr Michael Collins (Auckland City Hospital)*

*Professor Steve Chadban (University of Sydney)*

| Sponsor | **The Australasian Kidney Trials Network**  University of Queensland |
| --- | --- |
| Ethics committee reference | 17/NTA/62 |
| Trial registration number | ACTRN12617000358347 |
| Locality | XX |
| Site Principal Investigator | **XX**  Phone: XX  Email: XX |
| Research Coordinator | **XX**  Phone: XX  Email: XX |

**You are invited** to take part in the BEST-Fluids study. This study will test two types of intravenous (IV) fluids (given into a vein) in patients having a kidney transplant from a deceased organ donor. *Everyone who gets a transplant needs IV fluids and we are trying to find out the best type of fluid to use.*

**Whether or not you take part is your choice**. If you don’t want to take part, you don’t have to give a reason, and it won’t affect the care you receive. If you agree to take part, but change your mind later, you can pull out of the study at any time.

This **Participant Information Sheet** will help you decide if you’d like to take part. It sets out why we are doing the study, what your participation would involve, what the benefits and risks to you might be, and what would happen after the study ends. We will go through this information with you and answer any questions you may have. Because you need to have IV fluids during surgery, you will need to decide whether you will participate in this study BEFORE you go to the operating theatre for your transplant. We will give you as much time as possible to decide about participating. Before you decide, you may want to talk about the study with other people, such as family, whānau, friends, or healthcare providers. Feel free to do this.

If you agree to take part in this study, you will sign the **Consent Form** on the last page of this document. The study team will give you a copy of this Participant Information Sheet and the Consent Form to keep.

This document is 11 pages long, including the Consent Form. Please make sure you have read and understood all the pages.

#### What is the purpose of the study?

Everyone who has a kidney transplant needs IV fluid during surgery and for a few days afterwards. IV fluids contain water and a mixture of salts. They are important because they help:

- Maintain blood pressure, and
- Ensure good blood flow into the new kidney.

About one third of patients who have a kidney transplant from a deceased donor will have a delay for a period of time before their new kidney starts working. These patients often need dialysis to stay healthy while waiting for the new kidney to recover. This process usually takes a few weeks. Most transplant kidneys will return to normal function. However, this delay in kidney function can cause problems with recovery from surgery and may cause permanent damage to the new kidney.

Most hospitals, including XX Hospital, give kidney transplant patients an IV fluid called “Normal Saline”. This is a mixture of sodium chloride (salt) and water. Other names for Normal Saline include:

- 0.9% saline,
- Isotonic sodium chloride, or
- Simply ‘saline’.

Doctors have used Normal Saline in patients having surgery for many years. It is considered a safe fluid treatment. However, Normal Saline contains a large amount of chloride (part of what makes up salt) compared with blood and other body fluids. This can lead to increased levels of acid and potassium in the blood and might affect how well the new kidney functions after surgery.

In this study, we will compare Normal Saline with a low chloride IV fluid called Plasma-Lyte® 148 (Plasmalyte). *Plasmalyte* is a mixture of different types of salts designed to be more similar to normal body fluids than Normal Saline. Research in intensive care and surgical patients, including in kidney transplant patients, suggests that Plasmalyte might improve kidney function, compared with Normal Saline. However, the salts in Plasmalyte include potassium. This might lead to higher potassium levels in some kidney transplant patients.

Both Normal Saline and Plasmalyte are standard fluids given routinely to surgical patients. **The New Zealand Medicines and Medical Devices Safety Authority** (**MEDSAFE) and the Therapeutic Goods Administration (TGA) in Australia have approved both fluids for use in these patients.**

Currently, doctors are not sure which of these two fluids are better for kidney transplant patients. Even if you do not participate in this study, you might receive either or both of these fluids during and after your kidney transplant. This will depend on your doctor’s preferences. The aim of this study is to find out which fluid is better.

The results of this study will tell doctors:

- Which is the safest fluid;
- Which is most effective to support kidney function;
- Which will reduce the need for dialysis after transplant.

All patients who have a kidney transplant need IV fluids. This study is likely to influence the care of kidney transplant patients around the world.

This study is led and run by doctors specialising in kidney disease and kidney transplant from both Australia and New Zealand. Funding for the study is provided by public grants. The sponsor is the *Australasian Kidney Trials Network (AKTN; web address* [*www.aktn.org.au*](http://www.aktn.org.au)*)*. No commercial companies have provided funding support for this trial. Some of the fluids used in the study have been supplied via an unrestricted grant to the study team by Baxter Healthcare Australia Pty Ltd; Baxter have no other role in this study.

This study has been reviewed and received ethical approval from the Northern A Health and Disability Ethics Committee.

#### What will my participation in the study involve?

One of your transplant team will explain everything involved in the study. We will give you time to:

- Ask any questions you have,
- Discuss the study with family, whānau, friends, support people, or other healthcare providers, and
- Decide about participating.

You must make a decision BEFORE you go to the operating theatre for surgery. If you agree to participate, you will sign this consent form. We will give you a copy of your signed form to keep.

The study team will randomly allocate you by computer to receive either **Plasmalyte** or **Normal Saline** before, during and after your kidney transplant. By random, we mean that the allocation occurs by chance, a bit like tossing a coin. You will be blinded (unaware) to the type of fluid you have been allocated to. This means that neither you, nor your transplant doctors, nor the study team will know to which fluid you will receive. The fluids you receive will have a label with a code unique to you; this means that no one will be able to tell which type of fluid you get. However, if there is an emergency, your transplant doctor will be able to access this information.

It is important that the fluid you receive is allocated randomly and you are blinded to that fluid. This ensures that

- Researchers interpret the results in a fair and appropriate way, and
- Study doctors or participants don’t jump to conclusions.

You will receive the study fluid (Plasmalyte or Normal Saline) until 48 hours after your transplant operation. Your transplant doctors will decide the amount of study fluid you need, and may stop the fluid earlier than 48 hours after surgery if you no longer need IV fluids. If your doctors decide that you need a specific type of intravenous fluid, including Plasmalyte or Normal Saline, they will give this to you.

For all other treatments, you receive standard care for your kidney transplant. At XX Hospital, your care will follow the approved Renal Transplant Protocol.

You will be followed in the study for at least 12 months after your transplant surgery. **This will involve no extra visits to the hospital or outpatient clinics**. The study team will collect all study information at your usual hospital/clinic visits.

The BEST-Fluids study aims to include 800 kidney transplant patients in the study. Patients will come from transplant units in Australia and New Zealand.

#### What information will be collected about me?

You will be followed in the study from when you signed this form until 12 months after your transplant surgery. The study team will collect information about your health and kidney transplant from your hospital records. There is no need for extra visits or tests.

This information includes:

- Relevant personal details (age, gender, ethnicity, height, weight),
- Your medical history,
- Details about your kidney transplant,
- Whether you have dialysis after transplant,
- Blood test results that assess your kidney transplant function.

The study team will also collect information about your donor. You will not have access to this information to protect the privacy of the organ donor and their family.

If you have any medical scans just after your transplant, we will collect and store copies of any images or reports. This imaging may include ultrasounds and/or nuclear medicine scans. These scans will give the study team information about your kidney function. Collection of scans is optional. You can still be involved in the study if you do not agree to have your scans collected by the study team.

The study team will collect information about you at the following time points during the study:

- At admission for transplant surgery (Baseline)
- At arrival to recovery room after transplant surgery (Post-Op)
- 1 day after transplant (Day 1)
- 2 days after transplant (Day 2)
- 7 days after transplant (Day 7)
- 1, 3, 6 and 12 months after transplant (Month 1, 3, 6 and 12)

You will complete a short questionnaire about your health at some of these time points (Baseline, Day 7, and Month 1, 3, 6 and 12). The questionnaire will take about 5-10 minutes to complete and can be done while you are waiting to see your doctor at the clinic, or can be completed over the phone.

During the study, we will also collect information about your use of healthcare including:

- Any admissions to hospital
- Doctors’ visits
- Your medications
- Any medical tests

This information is stored with the NZ Ministry of Health. We will collect it using your unique National Health Index (NHI) number. This information will allow us to calculate the cost of your healthcare over 1 year. Any information about you will remain confidential and will only be used for the purposes we have told you about.

#### How will my information be stored, and who will have access to it?

The study team at XX Hospital will ensure all your information is stored securely. This information will be stored on site, or at selected storage location.

The study will use the ANZDATA registry to collect all patient information. ANZDATA stands for **Australia and New Zealand Dialysis and Transplant Registry**.

The ANZDATA registry focuses on dialysis and kidney transplant patients in Australia and New Zealand and

- Has been collecting information since the 1960’s;
- Publishes regular reports about treatment and outcomes of these patients
- Works with hospitals, doctors and researchers with an aim to improve patient outcomes;
- Is funded by the New Zealand and Australian Governments, and Kidney Health Australia.

The BEST-Fluids study will use some of the information normally collected by ANZDATA, and some extra information specific for this trial.

You may have already heard about ANZDATA. If you are on dialysis or had a transplant previously, your renal team may have already asked for permission to collect your information for ANZDATA. **By consenting to participate in this study, you are agreeing to have your information sent to ANZDATA**. *If you do not consent to have your health information sent to ANZDATA, you will not be able to participate in this study.*

All information recorded in ANZDATA is confidential. The identity of people in the database IS NOT released publicly nor in any reports. There are measures in place to ensure the security of all collected information.

The study team will have access to study information, where required for approved purposes. The Study team includes:

- Local study staff at XX Hospital,
- Designated staff at the AKTN, based at the University of Queensland,
- Staff at the ANZDATA registry, and
- Local regulatory bodies and responsible Ethics Committees.

Confidentiality of information will be maintained at all times.

#### What are the possible benefits and risks of this study?

Benefits

Taking part in this study may or may not make your health better compared to receiving your routine kidney transplant medical care. We expect that the information from this study will help doctors learn more about managing kidney transplant patients. This information could help other patients with kidney disease in the future.

Risks

Both of the fluid types used in this study are already in standard use in hospitals. Normal Saline is the most commonly used treatment for kidney transplant patients, but Plasmalyte is used at some centres. Based on current knowledge about these two fluid types, **the risks of participation in this study are low**. If you take part in this study, you will face similar risks to a kidney transplant patient who did not participate in this study. *Remember that having a kidney transplant can be risky. Your doctor should discuss any risks in detail with you as part of the process of your informed consent to have a kidney transplant.*

This study may involve unknown or unforeseen side effects of the study fluids. Both fluids have been given to a very large number of patients worldwide so unknown effects are unlikely.

Medical treatments often cause side effects. You may have none, some or all of the effects listed below, and they may be mild, moderate or severe. If you have any of these side effects, or are worried about them, talk with your transplant doctor or the study team. Your doctor will also be looking out for side effects. Many side effects go away shortly after treatment ends. However, sometimes side effects can be serious, long lasting or permanent. If a severe side effect or reaction occurs, your doctor may need to stop your treatment. Tell your doctor if you have any problems. Your doctor will discuss the best way of managing any side effects with you.

**Known side effects** of Normal Saline and Plasmalyte solutions as part of fluid treatment for kidney transplantation may include the following:

- Swelling of the hands, ankles and feet due to retention of fluid in your body. Rarely, this may involve pressure on the heart or the lungs, which may cause some breathing difficulty.
- High levels of potassium in your blood (hyperkalaemia) which can occasionally cause problems with your heart rate and rhythm. You might need to have urgent dialysis treatment.
- Disturbances in the level of acid or electrolytes in the body, e.g. high or low sodium levels (Hyper/hyponatraemia). This should be brief but might require a change to the type or amount of IV fluids you are given, or occasionally the use of medications.

Your transplant doctor can stop the study fluids if the study fluids are causing or worsening these problems and they believe stopping fluids is necessary for your ongoing care. High potassium levels are a known problem after kidney transplantation and can require treatment. For this reason, all patients have frequent monitoring of electrolyte and potassium levels, including at least once each day in the first week after transplant surgery.

It is also possible to have an allergic reaction to either of the fluids. However, this is extremely rare.

#### Who pays for the study?

There is no cost to participants to be involved in this study. No patients will receive any reimbursements or payments as part of this study.

The BEST-Fluids study is funded by

- The Royal Australasian College of Physicians (a *Jacquot Research Establishment Fellowship* awarded to Dr Michael Collins),
- The Health Research Council of New Zealand (a 2017 Project Grant)*,* and
- The Medical Research Future Fund in Australia
- The *Better Evidence And Translation in Chronic Kidney Disease (BEAT-CKD)* program in Australia, and
- A Baxter Investigator Initiated Research Grant to supply the study fluids (Baxter Healthcare).

#### What if something goes wrong?

If you were injured in this study, which is unlikely, you would be eligible **to apply** for compensation from the Accident Compensation Corporation (ACC) just as you would be if you were injured in an accident at work or at home. This does not mean that your claim will automatically be accepted. You will have to lodge a claim with ACC, which may take some time to assess. If your claim is accepted, you will receive funding to assist in your recovery.

If you have private health or life insurance, you may wish to check with your insurer that taking part in this study won’t affect your cover.

#### What are my rights?

Your participation is voluntary (your choice). You do not have to take part in this study, and if you choose not to take part, you will receive the usual standard care for your kidney transplant. If you do agree to take part, you are free to withdraw from the study at any time, without having to give a reason and this will in no way affect your future health care.

You have the right to access information about you collected as part of the study. Please ask one of the study team if you wish to do this.

You will be told of any new information about good or bad effects related to the study that may have an impact on your health.

No material which could personally identify you will be used in any reports on this study.

#### What happens after the study or if I change my mind?

Information collected as part of this study will be stored for a minimum of 15 years.

If you change your mind and decide to withdraw from (leave) this study, please let someone from the study team know. You do not have to give a reason, and it will not affect the medical care you receive. We may ask you for your consent to continue to collect some information about you following your withdrawal from the study. However, you do not have to agree to this if you do not wish to.

It is expected that the results of the study will be presented at medical conferences and published in medical journals, but there may be a gap of several years between the trial finishing and publication. **If you wish, you can be sent a summary of the results of the study when it is completed** – there is a box to request this on the consent form.

#### Who can I contact for more information or if I have concerns?

If you have any questions, concerns or complaints about the study at any stage, you can contact:

**The *BEST-Fluids Study* Research Coordinator**

- Phone: XX
- email: XX

**The *BEST-Fluids Study* Principal Investigator – Dr XX**

- Phone: XX
- email: XX

If you want to talk to someone who isn’t involved with the study, you can contact an independent health and disability advocate on:

Telephone (NZ wide): 0800 555 050

Free Fax (NZ wide): 0800 2787 7678 (0800 2 SUPPORT)

Email (NZ wide): advocacy@hdc.org.nz

If you require Māori cultural support, talk to your whanau in the first instance. Alternatively you may contact the administrator for He Kamaka Waiora (Māori Health Team) by telephoning 09 4868920 extension 2324.

If you have any questions or complaints about the study you may contact the Auckland and Waitemata District Health Boards Māori Research Committee or Māori Research Advisor by telephoning 09 4868920 extension 3204.

You can also contact the health and disability ethics committee (HDEC) that approved this study on:

Phone: 0800 4 ETHICS

Email: [hdecs@moh.govt.nz](mailto:hdecs@moh.govt.nz)

***Thank you for considering taking part.***

| **Consent Form** | Your letterhead |
| --- | --- |


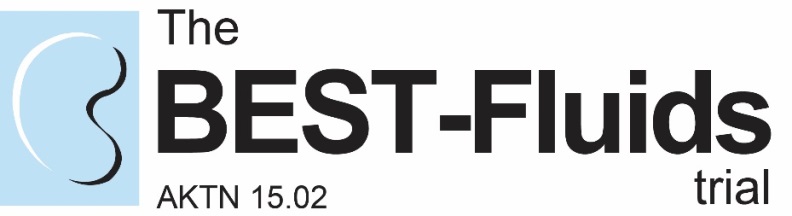


***B***etter ***E***vidence for ***S***electing ***T***ransplant ***Fluids***

An investigator-initiated, pragmatic, registry-based, multi-centre, double-blind, randomised controlled trial evaluating the effect of Plasmalyte versus 0.9% saline on early kidney transplant function in deceased donor kidney transplantation.

**If you need an INTERPRETER, please tell us.**

*An interpreter is available on request*

***I understand and agree with the following statements:***

| I have read, or have had read to me in my first language, and I understand the Participant Information Sheet. | | |
| --- | --- | --- |
| I have been given sufficient time to consider whether or not to participate in this study. | | |
| I have had the opportunity to use a legal representative, whanau/ family support or a friend to help me ask questions and understand the study. | | |
| I am satisfied with the answers I have been given regarding the study and I have a copy of this consent form and information sheet. | | |
| I understand that taking part in this study is voluntary (my choice) and that I may withdraw from the study at any time without this affecting my medical care. | | |
| I consent to the research staff collecting and processing my information, including information about my health. | | |
| I agree to an approved auditor appointed by the New Zealand Health and Disability Ethic Committees, or any relevant regulatory authority or their approved representative reviewing my relevant medical records for the sole purpose of checking the accuracy of the information recorded for the study. | | |
| I understand that my participation in this study is confidential and that no material, which could identify me personally, will be used in any reports on this study. | | |
| I understand the compensation provisions in case of injury during the study. | | |
| I know who to contact if I have any questions about the study in general. | | |
| I understand my responsibilities as a study participant. | | |
| If I decide to withdraw from the study, I agree that the information collected about me up to the point when I withdraw may continue to be processed. | | |
| I consent to my GP or current provider being informed about my participation in the study and of any significant abnormal results obtained during the study. | | |
| I consent to information and electronic data from **medical imaging tests** (ultrasound and nuclear medicine studies) taken as part of my care, including images and reports, being collected and processed for this study. | Yes 🞏 | No 🞏 |
| I wish to receive a summary of the results from the study. | Yes 🞏 | No 🞏 |

**Declaration by participant:**

I hereby consent to take part in this study.

| Participant’s name: | |
| --- | --- |
| Signature: | Date: |

**Declaration by member of research team:**

I have given a verbal explanation of the research project to the participant, and have answered the participant’s questions about it.

I believe that the participant understands the study and has given informed consent to participate.

| Researcher’s name: | |
| --- | --- |
| Signature: | Date: |
